# A Novel Mechanism of Salvianolic Acid B in Postmyocardial Infarction Cardiac Protection: PHB1‐Driven Raf‐ERK Pathway Activation Promotes Cardiomyocyte Mitosis

**DOI:** 10.1002/mco2.70752

**Published:** 2026-05-03

**Authors:** Ce Cao, Bo Ma, Jian Zhang, Lili Yang, Zixin Liu, Haoran Li, Jianshu Song, Keer Zhang, Lei Li, Jianhua Fu, Jianxun Liu

**Affiliations:** ^1^ National Clinical Research Center for Chinese Medicine Cardiology Beijing Key Laboratory of Chinese Materia Pharmacology Xiyuan Hospital of China Academy of Chinese Medical Sciences Beijing China; ^2^ State Key Laboratory of Bioactive Substance and Function of Natural Medicines Institute of Materia Medica Chinese Academy of Medical Sciences and Peking Union Medical College Beijing China; ^3^ Department of Critical Care Medicine Meizhou Hospital of Guangzhou University of Chinese Medicine Meizhou China; ^4^ Second Clinical College Guangzhou University of Chinese Medicine Guangzhou China

**Keywords:** cell cycle, cardiomyocyte proliferation, Prohibitin 1, Raf‐ERK pathway, salvianolic acid B

## Abstract

Heart failure (HF) following myocardial infarction remains a leading cause of global morbidity and mortality, necessitating novel therapeutic strategies. Salvianolic acid B (SalB), one of the major active components of *Salvia miltiorrhiza* Bunge, has been shown to effectively reverse ischemia‐induced myocardial infarction and improve cardiac function. Here, we report a novel mechanism by which SalB treats postmyocardial infarction heart failure by promoting cardiomyocyte re‐entry into the cell cycle. A novel protein target of SalB, Prohibitin 1 (PHB1), was identified using chemical biology techniques. Experiments involving overexpression and knockdown of PHB1 have demonstrated that SalB promotes cardiomyocyte mitosis by acting on PHB1, which in turn upregulates the phosphorylation level of the Raf‐ERK pathway. Molecular dynamics simulations provide a comprehensive explanation for the mechanism by which SalB enhances Raf phosphorylation. The findings reveal that SalB binds to the C‐terminal of PHB1/2 heterodimer, inducing a conformational change that enhances Raf‐ERK pathway activation via Akt‐mediated phosphorylation, thereby promoting cardiomyocyte mitosis. The findings of this study propose a promising new molecular mechanism through which SalB can contribute to the preservation and restoration of cardiac function.

## Graphical abstract

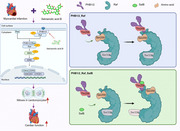

## 1 Introduction

Postmyocardial infarction heart failure (p‐MIHF) is a prevalent complication of myocardial infarction (MI), with mortality rates strongly correlating with the severity of heart failure (HF) [1, 2]. In the early stages of the disease, coronary blood flow is drastically reduced or interrupted for various reasons, resulting in localized myocardial ischemic necrosis [3, 4]. Myocardial cell necrosis is one of the key factors in coronary artery disease because when coronary arteries are narrowed or occluded, the heart is not adequately perfused with blood, resulting in myocardial cell ischemia and hypoxia, which in turn causes myocardial injury. As cardiomyocytes (CMs) are terminally differentiated cells, the heart is virtually incapable of regenerating new CMs. Consequently, damaged CMs are replaced by fibrous tissue, which leads to a decline in cardiac function.

The inability of cardiomyocytes to regenerate is primarily due to shifts in respiratory and energy metabolism patterns, maturation of the immune system, and the establishment of circadian rhythms [5, 6]. Previous studies have shown that activation and re‐entry of the cardiomyocyte cycle are critical steps in endogenous myocardial neogenesis [5, 7]. Studies in various animal models showed that residual CMs in damaged hearts acquire proliferative capacity by modifying cell‐cycle‐related genes or regulating growth‐related signals. Different physiological and pathological environments, such as hypoxic environments, oxygen‐rich environments, and immune responses, are actively involved in cardiomyocyte neogenesis [8, 9]. The Prohibitin (PHB)‐mitogen‐activated protein kinase (MAPK)‐extracellular regulated protein kinases (ERK) pathway plays a crucial role in regulating the cell cycle. Previous studies have shown that activation of the rat sarcoma (Ras)‐induced rapidly accelerated fibrosarcoma (Raf)‐mitogen‐activated extracellular signal‐regulated kinase (MEK)‐ERK pathway is dependent on PHBs. Phosphorylated PHBs first bind to Raf to form a dimer, which is then recruited by guanosine triphosphate (GTP)‐bound Ras proteins to form a multimer in the cell membrane, which in turn activates the Raf‐MEK‐ERK pathway. Subsequently, phosphorylated ERK translocases to the nucleus and initiates transcription of the cell cycle proteins cyclin dependent kinase (CDK) ‐ 4 (CDK4), CDK6, cell division cycle 25 (CDC25) and Cyclin D, which promotes cell cycle re‐entry, enhances RNA and protein biosynthesis in G_1_ phase and re‐initiates mitotic activity of cells, ultimately promoting cellular neogenesis [10, 11, 12, 13]. However, the PHB‐MAPK‐ERK pathway in cardiomyocytes is relatively understudied and needs to be further investigated.

As a traditional Chinese medicine (TCM), *Salvia miltiorrhiza* Bunge has been used in the treatment of cardiovascular diseases for thousands of years. Nowadays, pharmacological studies show that *S. miltiorrhiza* Bunge has the functions of antioxidant, anti‐inflammatory, CMs protection, endothelial cell protection, promotion of angiogenesis, regulation of vasodilation, and anti‐atherosclerosis [14, 15, 16, 17, 18]. Salvianolic acid B (SalB), as the main active ingredient of *S. miltiorrhiza* Bunge, has been found to have important medicinal properties in reducing heart damage, antioxidant, antifibrotic, and antitumor properties [19, 20, 21, 22, 23, 24]. These studies have shown that SalB plays an important role in cardioprotection, but have not explained the targets and mechanisms of its action on the cell cycle [25, 26].

In this study, we identified SalB's target protein PHB1 in CMs and found that SalB activates the cell cycle by modulating the local conformation of the PHB1 protein. When SalB binds to the C‐terminus of the PHB heterodimer, its binding mode with Raf undergoes a significant conformational change, enabling a closer binding between PHB1 and the Raf protein. This enhances the recognition and phosphorylation of Raf by protein kinase B (Akt), thereby activating the Raf‐ERK signaling pathway and promoting the process of cell mitosis.

## 2 Results

### 2.1 Salvianolic Acid B Promotes Cardiomyocyte Mitosis in Postmyocardial Infarction Heart Failure

**FIGURE 1:**
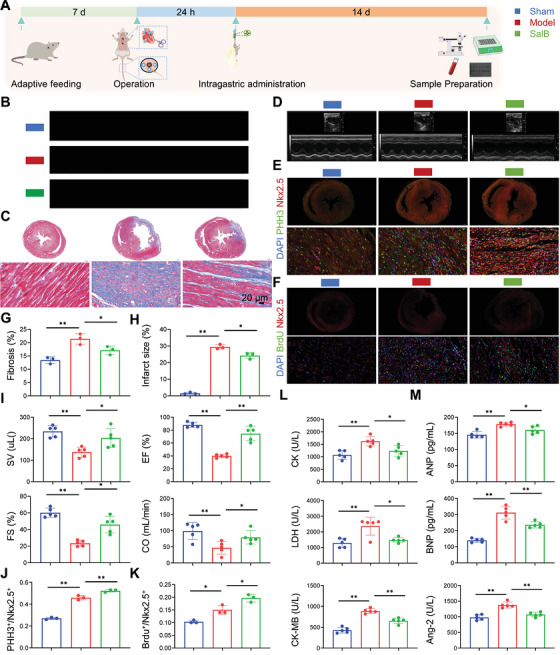
Salvianolic acid B promotes cardiomyocyte proliferation in postmyocardial infarction heart failure. (A) Schematic of in vivo animal experimental design. (B) Representative images of infarct size in each group. (C) Representative images of Masson staining in each group. (D) Representative images of echocardiographic findings in each group. (E) Representative images of immunofluorescence staining for detecting mitotic cardiomyocytes. (F) Representative images of immunofluorescence staining for detecting regenerating cardiomyocytes. (G) Quantitative analysis of Masson staining in each group (*n* = 3). (H) Quantitative analysis of infarct size in each group (*n* = 3). (I) Quantitative analysis of echocardiographic parameters in each group (*n* = 5). (J) Quantitative analysis of immunofluorescence for mitotic cardiomyocytes (*n* = 3). (K) Quantitative analysis of immunofluorescence for regenerating cardiomyocytes (*n* = 3). (L) Levels of CK, CK‐MB, and LDH in rat serum (*n* = 5). (M) Levels of ANP, BNP, and Ang‐2 in rat serum (*n* = 5). PHH3 marks mitotic cells, Nkx2.5 marks cardiomyocytes and BrdU marks regenerating cells.

After 7 days of adaptive feeding, rats underwent coronary artery ligation of the left anterior descending branch. SalB was given by intragastric administration, and BrdU was injected intraperitoneally starting 24 h after the operation for 2 weeks (Figure 1A). Compared with the sham group, model had significantly increased the infarct size (Figure 1B,H), larger fibrosis (Figure 1C,G), decreased stroke volume (SV), fractional shortening (FS), ejection fraction (EF), cardiac output (CO) (Figure 1D,I), higher expression of creatine kinase (CK), creatine kinase‐MB Isoenzyme (CK‐MB), lactate dehydrogenase (LDH), atrial natriuretic peptide (ANP), B‐type natriuretic Peptide (BNP) and angiopoietin‐2 (Ang‐2) (Figure 1L,M). As expected, SalB treatment could regress the remodeling‐associated changes in these rats. Meanwhile, we used immunofluorescence to detect mitosis and neogenesis in CMs. Results showed ligation of the left anterior descending branch of the coronary artery significantly increased mitosis and neogenesis of CMs compared with the sham group, while administration of SalB treatment further increased this phenomenon (Figure 1E,F,J,K). These results further confirmed that SalB promoted cardiomyocyte proliferation in postmyocardial infarction heart failure. This interesting phenomenon prompted us to further explore the specific mechanism by which SalB promotes myocardial proliferation.

### 2.2 SalB Significantly Improves Cell Viability and Decreases Cell Apoptosis

**FIGURE 2:**
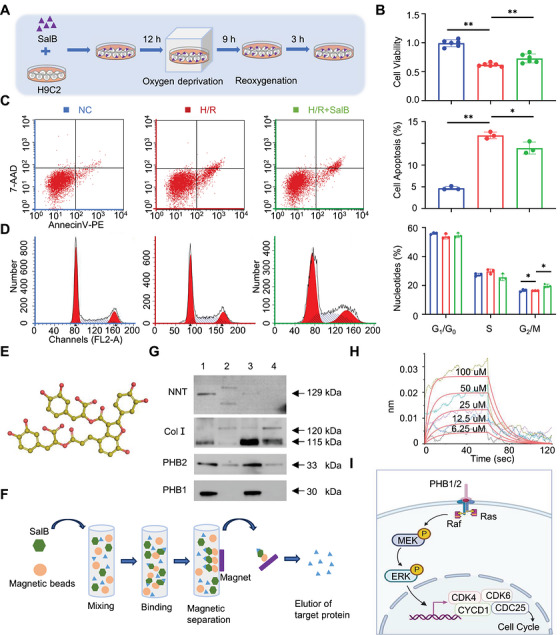
SalB significantly enhances cell viability, reduces apoptosis, and directly interacts with PHB1 to alter the cell cycle in H/R‐induced H9C2 cells. (A) Schematic diagram of the process of cell culture. (B) Cell viability of H9C2 with SalB after H/R (*n* = 6). (C) Cell apoptosis of H9C2 with SalB after H/R (*n* = 3). (D) Nucleotides percentage of H9C2 with SalB after H/R (*n* = 3). (E) Chemical structure of Salvianolic acid B. (F) Schematic diagram of a Salvianolic acid B coupling to magnetic beads. (G) Western blot detection of NNT, Collagen 1, Prohibitin 2, and Prohibitin 1 protein expression by magnetic bead‐immunoprecipitation assays (From left to right, total protein, BSA control, magnetic bead precipitate, magnetic bead supernatant). (H) Biolayer interferometry of salvianolic acid B and Prohibitin 1. (I) Identification of key signaling pathways for Prohibitin 1 after reviewing the literature.

Using an in vitro model of CMs, we first investigated the effects of SalB. SalB and H9C2 cells were co‐cultured for 24 h, followed by 9 h of oxygen deprivation and 3 h of reoxygenation (Figure 2A). As shown in Figure 2B,C, hypoxia/reoxygenation (H/R) significantly decreased cell viability and decreased cell apoptosis when compared with the normal control (NC) group. Administration of SalB attenuated the damage caused by hypoxia‐reoxygenation to H9C2 cells, indicating that SalB has a significant protective effect on CMs. Interestingly, we also found that SalB can influence the G_2_/M of the cell cycle in H9C2 after H/R, suggesting that SalB may promote rapid cell growth by facilitating more cells to enter the mitotic preparatory and mitotic phases, thereby improving the survival of injured cells (Figure 2D).

### 2.3 Identification of Prohibitin 1 as a Direct Target of Salvianolic Acid B to Regulate Cell Cycle

To investigate the target protein of the effect of SalB on H9C2, we first carried out magnetic bead precipitation assays. Suitable magnetic beads were selected to produce magnetic beads‐SalB (Figure 2E). H9C2 cells were cultured in the presence of magnetic beads alone or magnetic beads‐SalB (Figure 2F). Magnetic beads conjugated with SalB exhibited significant aggregation around H9C2 cells, whereas unconjugated beads showed no binding. To verify the binding of magnetic beads‐SalB to H9C2 cells, we extracted the total protein and stained it with Kaomas brilliant blue, and observed that SalB binds to numerous proteins (Figure S1). Based on mass spectrometry analysis, we found SalB may bind nicotinamide nucleotide transhydrogenase (NNT), Collagen 1, PHB1, and PHB2. We further investigated the interactions of SalB with NNT, Collagen 1, PHB1, and PHB2. High levels of PHB1 were detected in the proteins isolated from magnetic bead precipitation samples, while NNT was not detected. Collagen 1 and PHB2 were detected in the proteins isolated from magnetic bead precipitation and supernatant samples, but at low levels, which showed that SalB could directly bind PHB1 and some levels of PHB2, but could not bind NNT proteins (Figure 2G). In addition, we examined the binding of SalB to PHB1 using bio‐layer interferometry (BLI), and the two combined with an affinity of 4.75E‐05 (KD value), suggesting that SalB exerts its pharmacodynamic effects through direct action on PHB1 (Figure 2H).

### 2.4 PHB1 Regulates the MEK/ERK Signaling Pathway to Influence Cell Cycle and Promote Cell Survival

**FIGURE 3:**
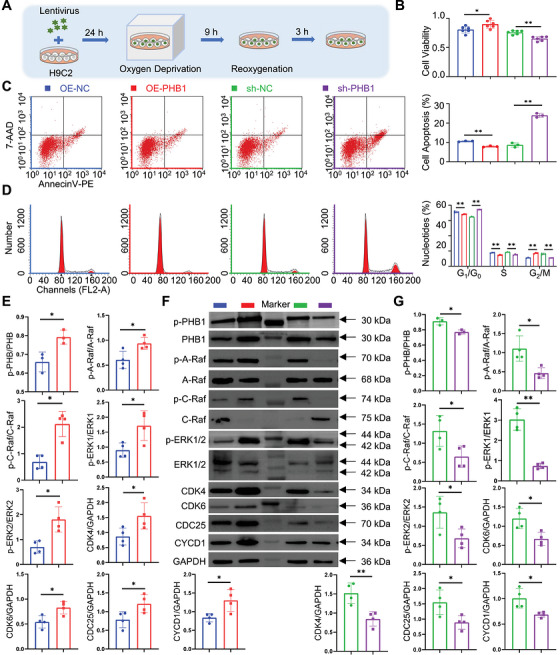
PHB1/MEK/ERK signaling pathway regulates cell cycle in H9C2. (A) Schematic diagram of lentivirus and cell co‐culture process. (B) Cell viability of H9C2 with lentivirus (*n* = 6). (C) Cell apoptosis of H9C2 with lentivirus (*n* = 3). (D) Nucleotides percentage of H9C2 with lentivirus (*n* = 3). (E) The changes in the expression of signaling pathway proteins after overexpression of Prohibitin1 (*n* = 4) (F) Typical images of western blots. (G) The changes in the expression of signaling pathway proteins after knocking down Prohibitin1 (*n* = 4).

PHB1 plays an important role in the cell cycle. PHB1 and PHB2 can bind to Raf, Ras, and subsequently activate the MEK/ERK signaling pathway, which ultimately affects the cell cycle (Figure 2I). Thus, SalB may act directly on PHB1 and activate the MEK/ERK signaling pathway in H9C2 cells, ultimately affecting the cell cycle of CMs. To test this hypothesis, we first investigated the role of PHB1 on the MEK/ERK pathway and the cell cycle of H9C2, and we constructed lentiviral knockdown and overexpression of PHB1 in the H9C2 cell line (Figure 3A). Comparative experiments showed that overexpression of PHB1 leads to a corresponding increase in protein expression of the MEK/ERK pathway and increases cell viability and reduces apoptosis (Figure 3B,C). In contrast, PHB1 knockdown had the opposite effect. In particular, flow cytometric sorting of the cell cycle showed that overexpression of PHB1 allowed more cells to enter the G_2_/M phase, whereas PHB1 knockdown significantly reduced the number of cells in the G_2_/M phase (Figure 3D). Western blot results showed the same trend (Figure 3E,F,G). A‐Raf, B‐Raf, and C‐Raf belong to the Raf family. All three are serine/threonine kinases that play a core role in the Ras‐Raf‐MEK‐ERK signaling pathway, regulating cell proliferation, differentiation, and survival processes. Among them, A‐Raf and C‐Raf are widely expressed and play key roles in various cell types; in contrast, B‐Raf is highly expressed primarily in neural tissues, and its activation process is independent of Prohibitin 1 (PHB1). Based on the above characteristics, B‐Raf was not detected in this study. The G_2_/M phase is important for cell survival because it can stimulate damaged cells to repair themselves and also to synthesize RNA and proteins in preparation for mitosis. These results further confirmed that PHB1 induces increased cell survival by regulating the MEK/ERK pathway, thereby affecting the cell cycle.

### 2.5 Human PHB1/MEK/ERK Signaling Pathway Trends Consistent With Animal Experiments

To further define the function of PHB1 in the heart, we analyzed data from the GEO database of human cardiac transcriptome samples. This dataset (GEO ID: GSE141910) contained 160 nonfailing donors (Control), 22 peripartum cardiomyopathy (PPCM), 164 dilated cardiomyopathy (DCM), and 7 hypertrophic cardiomyopathy (HCM). The results showed that most of the levels of PHB and its related proteins were reduced in the hearts of DCM and HCM patients compared with patients without HF (Figure S2). PHB1 exhibits no significant downregulation in PPCM, which may be linked to the pathogenesis of PPCM. The disease is typically triggered by the combined effects of hormonal fluctuations, inflammatory responses, and oxidative stress during late pregnancy or the postpartum period, and this pathogenic mechanism likely endows myocardial cells with the capacity for reversible recovery. In contrast, PHB1 is significantly downregulated in patients with DCM and HCM. This phenomenon may be attributed to the fact that both DCM and HCM have myocardial dysfunction as their core pathological feature. Furthermore, downstream signaling pathway proteins of PHB1 (including Raf, ERK, CDK4, CDK6, and CDC25) are also significantly decreased in DCM and HCM. This finding indicates that the regenerative capacity of myocardial cells is significantly inhibited in these two subtypes. These results laterally corroborate that PHB expression trends are essentially the same in human and rat hearts after postmyocardial infarction HF.

### 2.6 SalB Directly Binds to PHB1 and Changes Its Structure

PHB1 forms a ternary complex by forming a heterodimer with PHB2 and binding Raf and Ras, which subsequently triggers Raf phosphorylation, thereby activating ERK to promote cell survival [12]. Thus, SalB binding to PHB1 may directly influence its binding to Raf and Ras.

**FIGURE 4:**
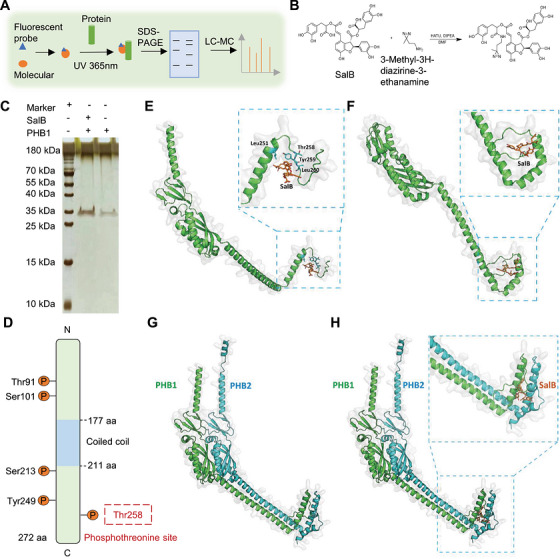
Determination of the protein‐bound forms of salvianolic acid B and Prohibitin 1. (A) Schematic diagram of activity‐based protein profiling. (B) Chemical Equation of activity‐based protein profiling. (C) SDS‐PAGE detection analysis. (D) Prohibitin1 protein sequence and binding site. (E) Molecular docking of Salvianolic acid B and Prohibitin1. (F) Molecular dynamics modeling of Salvianolic acid B and Prohibitin 1. (G) Molecular dynamics detection of Prohibitin 1/2 protein complex. (H) Molecular dynamics detection of the effect of salvianolic acid B on the Prohibitin 1/2 protein complex.

To understand the specific mechanism of SalB binding to PHB1 and activating downstream pathways, we combined SalB with 3‐methyl‐3H‐bisacridin‐3‐ethylamine to obtain probe‐labelled target compounds (Figure 4A,B). Liquid chromatography with tandem mass spectrometry (LC‐MS/MS) identified SalB binding to the THR‐258 site of PHB1 (Figure 4C,D). Meanwhile, we also obtained the structure of the complex of PHB1 with SalB by molecular docking (Figure 4E; Figure S3). The result showed that SalB binds within a pocket at the C‐terminus of the PHB1 with a binding energy of −5.3 kcal/mol, engaging interactions with Leu251, Thr258, Tyr259, and Leu260, and forming hydrogen bonds with Leu260, which was in complete agreement with the experimental results of the activity‐based protein profiling (ABPP) [27, 28]. Furthermore, we conducted molecular dynamics simulations on the PHB1‐SalB complex using the GROMACS program [29, 30]. Our findings revealed that during a 100‐nanosecond (ns) simulation, SalB stably binds to the C‐terminus of PHB1 (Figure 4F; Figure S4,5). As the simulation progressed, conformational changes in the C‐terminus of PHB1 led to alterations in the binding pocket of SalB. Nonetheless, SalB maintained stable binding between two helices at the C‐terminus. In fact, under physiological conditions, PHB1 forms a heterodimer with PHB2 and directly interacts with Raf through the C‐terminal region, subsequently binding and activating Ras and downstream pathways. Therefore, the interaction between PHB1 and SalB to some extent can demonstrate their stable binding, while the structural mechanism of the complex requires further consideration of the involvement of other proteins.

### 2.7 SalB Activates Downstream Pathways by Altering the Competitive Phosphorylation of PHB1 With Raf

In previous studies, it was found that PHB1 forms a heterodimer with PHB2 and activates Raf by directly binding to its C‐terminus [31]. Raf is in an inactive state when Ser259 is phosphorylated by Akt, and it is activated when Ser338 is phosphorylated under the stimulation of EGF. Additionally, Thr258 of PHB1 can also be phosphorylated by Akt. The PHB1‐Thr258 and Raf‐Ser259 are located in the same Akt recognition sequence R‐x‐R‐x‐x‐S/T, leading to a competitive inhibitory effect [31]. Therefore, SalB may regulate the phosphorylation of Raf and PHB1 by binding to the C‐terminus of PHB1/2, thereby regulating the activity of downstream pathways. To elucidate the structural mechanism by which SalB regulates the activation of Raf by binding to PHB1, we conducted further molecular dynamics simulations. Initially, we predicted the heterodimer structure of PHB1 and PHB2 using AlphaFold3 (Figure 4G). Subsequently, we docked SalB to the C‐terminus of the PHB1/2 dimer using the AutoDock Vina program, obtaining the PHB1/2_SalB complex structure (Figure 4H). Within the PHB1/2 dimer, PHB1 and PHB2 are arranged in a parallel stacking conformation, similar to the stacking pattern of HflK and HflC with PHB domains in prokaryotic cells [32]. In the PHB1/2_SalB complex, SalB binds to a pocket formed at the C‐terminus of the complex by both PHB1 and PHB2, with a binding free energy of −9.0 kcal/mol. SalB binds to the C‐terminal pocket of the PHB1/2 complex with higher affinity than PHB1 alone.

**FIGURE 5:**
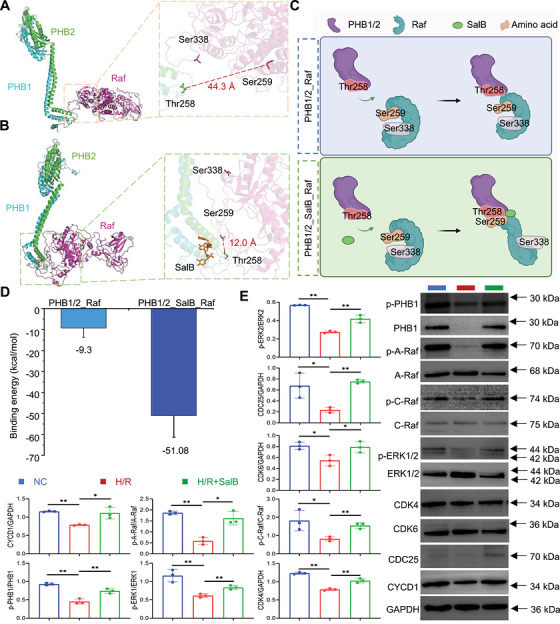
SalB activates downstream pathways by altering the competitive phosphorylation of PHB1 with Raf. (A) Binding of PHB1/2 complexes and Raf in the absence of salvianolic acid B. (B) Binding of PHB1/2 complexes and Raf upon incorporation of salvianolic acid. (C) SalB can alter the binding of Raf to PHB1/2 and form additional contacts. (D) Effect of SalB on the binding energy of the PHB1/2 complex bound to Raf. (E) Western blotting to verify the effect of Salvianolic acid B on PHB 1 and its pathway (*n* = 3). PHB, Prohibitin.

Previous studies have identified that PHB1 directly interacts with Raf protein through PHB1 (243–272) and Raf (297–335) [11, 33]. Based on this information, we utilized the Hdock program to perform molecular docking of the PHB1/2 complex and Raf, obtaining the complex structures of PHB1/2_Raf and PHB1/2_SalB_Raf (Figure S6). Subsequently, we conducted molecular dynamics simulations on these two complexes using the GROMACS program. Through 100 ns of simulation, we observed a significant shift in the binding conformation of Raf with PHB1/2 upon SalB binding (Figure 5A,B; Figure S7 and S8). In addition to the previously reported binding site, another flexible region of Raf (250‐270) also interacts with the PHB1/2 protein (Figure S9). The binding of SalB likely alters the conformation of the C‐terminus of the PHB1/2 protein, thereby altering the binding of Raf to PHB1/2 and forming additional contacts. This binding leads to a closer proximity between Raf‐Ser259 and PHB1‐Thr258 (from 44.3 to 12.0 Å), resulting in competitive inhibition of Raf‐Ser259 phosphorylation and facilitating the activation of Raf. Moreover, the change in binding angle exposes Raf‐Ser338, which is buried in the binding interface, on the protein surface, making it more accessible for phosphorylation by kinases (Figure 5C). Structural superposition of the C‐terminal helical regions (PHB1: Gly232–Ser254; PHB2: Asn245–Ser267) of the PHB1/2 complex before and after SalB binding revealed that the two helices align well, while the subsequent flexible regions (PHB1: Arg255–Gln272; PHB2: Gln268–Lys299) undergo significant conformational changes (Figure S10A). In addition, the angles between the helices and the protein main bodies (PHB1: Gly176–Gly232; PHB2: Ser190–Asn245) increase markedly upon SalB binding, changing from 86.06° and 93.35° in the apo state to 112.21° and 98.43° in the bound state, respectively (Figure S10B,C). In the absence of SalB, hydrogen bonds are formed between Gln269 and Gln272 in PHB1, and Val279, Leu282, and Asp292 in PHB2 with their respective helical segments. These interactions stabilize the C‐terminal region in a closed conformation, restricting the mobility of the flexible regions (Figure S11A). Upon SalB binding, notable conformational shifts occur in the segments PHB1 Gln264–Gln272 and PHB2 Asn281–Lys299, with only Gln272 in PHB1 maintaining a hydrogen bond with the helix (Figure S11B). The conformational rearrangement of the flexible regions, together with the helices, creates an open binding pocket for SalB, facilitating extensive interactions with the ligand (Figure S11C). These include hydrogen bonds with Glu237, Gly263, Ser265, and Val266 in PHB1, and Lys253 and Asn277 in PHB2, as well as hydrophobic contacts with Ala244 and Ile247 in PHB1 and Ile260 in PHB2. Furthermore, SalB binding induces a pronounced increase in the angle between the C‐terminal helix and the protein core, particularly in PHB1, which consequently modulates the interaction between the C‐terminal region and Raf. In the unbound state, Raf forms only one hydrogen bond with Gln225 in the PHB1 C‐terminal helix (Figure S11D). In contrast, SalB binding promotes multiple interactions between Raf and PHB1, including hydrogen bonds with Asp233, Asp246, Gln250, and Arg253 in the helical segment, as well as with Ser254, Leu268, Leu270, and Gln272 in the flexible region (Figure S11E,F). Moreover, the C‐terminal flexible region of Raf moves closer to SalB and becomes part of the ligand‐binding pocket. At the same time, we calculated the MMPBSA binding free energy of PHB1/2 and Raf before and after the combination with SalB, and found that SalB can increase the binding affinity between PHB1/2 and Raf by more than five times (Figure 5D). SalB can activate Raf phosphorylation via PHB1, which in turn activates downstream pathways, as demonstrated by in vitro western blot experiments (Figure 5E). These factors all contribute to the activation of Raf, which may be a novel mechanism by which SalB binds PHB1/2 and activates Raf.

### 2.8 PHB1 is Essential for SalB to Promote Cardiomyocyte Proliferation in Rat and Human Cardiomyocytes

**FIGURE 6:**
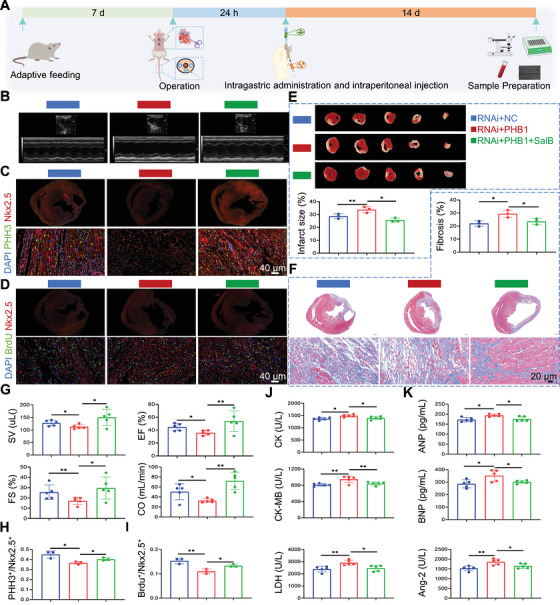
Salvianolic acid B promotes cardiomyocyte proliferation in post‐myocardial infarction heart failure via PHB1. (A) Schematic diagram of in vivo animal experiments. (B) Typical images of echocardiographic changes in each group. (C) Typical images of immunofluorescence assay for the detection of mitosis in cardiomyocytes. (D) Typical images of immunofluorescence assay for the detection of regeneration in cardiomyocytes. (E) Changes in infarct size in each group (*n* = 3). (F) Changes in Masson staining in each group (*n* = 3). (G) echocardiographic changes in each group (*n* = 5). (H) Immunofluorescence assay for detection of mitosis cardiomyocytes (*n* = 3). (I) Immunofluorescence assay for the detection of regenerating cardiomyocytes (*n* = 3). (J) Determination of CK, CK‐MB, and LDH in rat serum (*n* = 5). (K) Determination of ANP, BNP, and Ang‐2 in rat serum (*n* = 5). PHH3 marks mitotic cells, Nkx2.5 marks cardiomyocytes and BrdU marks regenerating cells.

**FIGURE 7:**
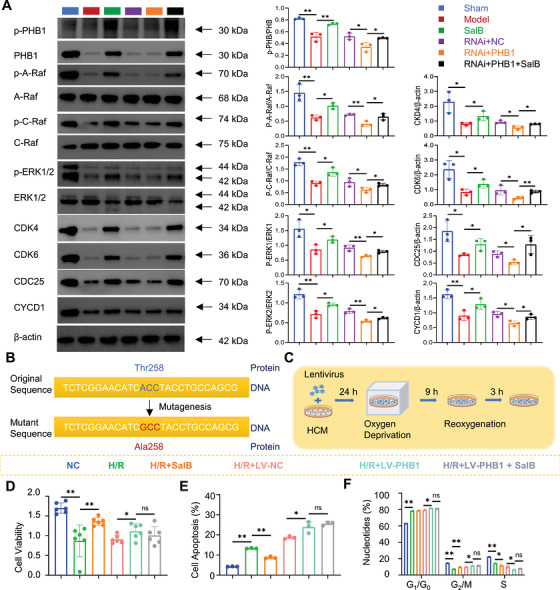
Salvianolic acid B promotes cardiomyocyte regeneration in post‐myocardial infarction heart failure by regulating PHB1. (A) Expression of PHB and pathways in post‐myocardial infarction heart failure (*n* = 3). (B) Diagram of lentivirus‐mediated point mutation of Prohibitin protein from Thr258 to Ala258 in human cardiomyocyte. (C) Schematic diagram of the process of cell culture. (D) Cell viability of human cardiomyocytes with SalB after H/R (*n =* 6). (E) Cell apoptosis of human cardiomyocytes with SalB after H/R (*n =* 3). (F) Nucleotides percentage of human cardiomyocyte with SalB after H/R (*n =* 3).

Considering that SalB could promote phosphorylation of PHB1 and activate Raf‐MEK‐ERK signaling in vitro, we further evaluated the impact of decreased PHB1 on the heart and the action of SalB in vivo. Adeno‐associated virus, RNAi+PHB1, driven by a specific cTNT promoter, was constructed and intravenously injected into the rat heart to induce cardiomyocyte‐specific under‐expression of PHB1. After 7 days of adaptive feeding, rats underwent coronary artery ligation of the left anterior descending branch and tail vein injection of adeno‐associated virus. SalB was given by intraperitoneal gavage, and BrdU was injected intraperitoneally starting 24 h after the operation for 2 weeks (Figure 6A). Compared with the RNAi+NC group, the RNAi+PHB1 group had significantly decreased SV, FS, EF, and CO (Figure 6B,G), increased the infarct size (Figure 6E), larger fibrosis (Figure 6F), and higher expression of CK, CK‐MB, LDH, ANP, BNP, and Ang‐2 (Figure 6J,K). Meanwhile, to assess the effects of underexpression of PHB1 on CMs and SalB treatment in vivo, we used immunofluorescence to detect mitosis and neogenesis in CMs. Underexpression of PHB1 on CMs decreased the number of mitoses and regenerating CMs (Figure 6C,D,H,I). As expected, these symptoms were significantly improved after 2 weeks of SalB treatment, supporting that SalB plays its role through PHB1. Western blot revealed that SalB could promote the activation of PHB1 and downstream proteins (Figure 7A; Figure S12–S15).

To further clarify the mechanism by which SalB exerts its protective effect against H/R injury by binding to the 258th site of PHB1 protein, we used lentivirus‐mediated site‐directed mutagenesis to mutate threonine at position 258 of PHB1 protein in human cardiomyocytes to alanine (Figure 7B). The experimental procedure is shown in Figure 7C, and the results demonstrated that SalB could effectively reverse H/R‐induced decrease in cell viability, increase in apoptosis, and significantly regulate cell cycle progression. However, when the 258th site of PHB was mutated, SalB could not bind to this mutant site, and thus its reversing effect on H/R injury was completely blocked (Figure 7D–F). Combined with the consistent results of signal pathway protein expression, this study clearly confirms that the 258th site of PHB1 protein is a key molecular target for SalB to exert its protective effect against H/R injury (Figure S16 and S17). These results further confirmed the critical role of the PHB1‐dependent Raf‐MEK‐ERK pathway in the action of PHB1 on myocardial proliferation.

## 3 Discussion

**FIGURE 8:**
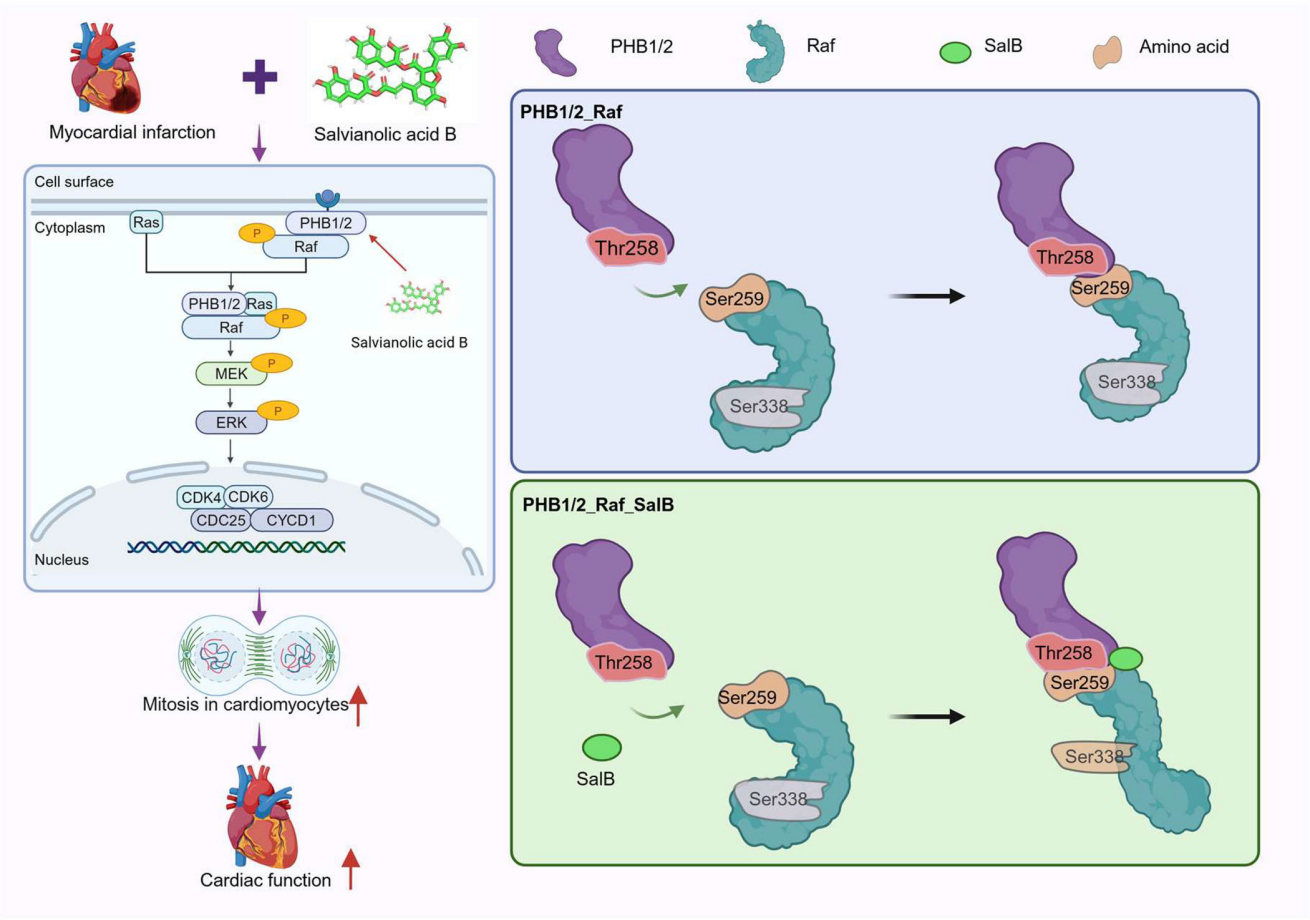
Salvianolic acid B promotes cardiomyocyte proliferation in postmyocardial infarction heart failure via Prohibitin 1.

In this study, we discovered that SalB, one of the main active ingredients of *S. miltiorrhiza* Bunge, reversed infarction‐induced adverse remodeling and HF by directly changing the cell cycle. A newly identified function of this compound is responsible for the proliferation of CMs. SalB specifically promoted the p‐MIHF effects of pathologically decreased CMs by interacting with PHB1 and activating the MEK/ERK signaling pathway. These experimental results revealed a novel mechanism for the proliferation of infarction‐induced CMs through targeted phosphorylation of PHB1 by SalB (Figure 8).

CMs necrosis is a key factor in coronary heart disease. When coronary arteries become narrowed or blocked, the heart is not adequately supplied with blood, leading to CMs ischemia and hypoxia, which in turn causes myocardial injury [3, 34, 35]. Adult CMs in the adult mammalian heart are widely recognized as terminally differentiated cells, typically arrested in the G_0_ phase of the cell cycle and exhibiting extremely limited proliferative potential. This intrinsic cellular characteristic is a key determinant of the markedly attenuated myocardial repair process following injury. It is difficult for the lost apoptotic myocardium to recover, and necrosis occurs, ultimately leading to the occurrence of various heart diseases such as myocardial infarction, malignant arrhythmia, HF, and sudden cardiac death [36]. Current pharmacological treatments and operations have been used to improve cardiac function, but have not yet solved the challenge of repairing and regenerating the heart. Recent studies have shown that the heart of newborn mammals can regenerate within a short time after birth, and that the regenerative capacity of the adult mammalian heart is limited, mainly due to the fact that the CMs cell cycle is arrested after birth [37, 38]. Over the past two decades, a large number of studies have explored the regulatory mechanisms of the cell cycle to enhance myocardial proliferation after myocardial infarction, which, by targeting cell cycle regulatory factors, can increase CMs mitosis, improve cardiac function, and improve long‐term survival [39, 40, 41, 42, 43, 44]. Generally, terminally differentiated adult cardiomyocytes can re‐enter the cell cycle and form new cardiomyocytes through a 3‐step process: dedifferentiation, proliferation, and redifferentiation [45]. Strikingly, SalB showed potent activity in directly changing the cell cycle both in vivo and in vitro, significantly ameliorating CMs’ death due to ischemia, suggesting that SalB is an attractive candidate drug for targeting to promote CMs proliferation.

Advances in magnetic bead ligand fishing technology have provided a platform for exploring new insights into the mechanisms of myocardial proliferation, particularly the mechanisms of TCM for improving HF [46]. Numerous targets have been identified in previous studies using magnetic bead ligand fishing [47, 48, 49]. These findings open up a promising area for HF drug development using magnetic bead ligand fishing as a research method to explore therapeutic targets in TCM. Proliferation of existing CMs requires dedifferentiation and subsequent re‐entry into the cell cycle. Growth factors such as PHB, bone morphogenetic proteins (BMPs), transforming growth factor (TGF), follicle suppressor‐like protein 1 (FSTL1), and proliferative miRNAs can lead to the activation of pro‐proliferative developmental pathways and/or influence the expression of cell cycle regulatory factors that promote CMs proliferation [50]. In contrast, transcription factors p53 or Meis homeobox 1 (MH1) lead to cell cycle arrest [50]. In addition, miRNAs can inhibit CMs’ proliferation by suppressing sarcomere disassembly and/or promoting cell cycle arrest [51]. However, how these TCM dynamically regulate and control cell cycle factors and their significance in human HF remain largely unknown. Our current study defined PHB1 as a novel key mechanism that promotes the progression of myocardial proliferation in the face of pathological stress in HF. SalB specifically promotes CMs mitosis and proliferation by enhancing PHB1 phosphorylation and promoting the Raf/MEK/ERK signaling pathway in CMs through interaction with PHB1, reversing cell cycle and HF. For the first time in the literature, we provide compelling evidence that targeting of pathologically downregulated PHB1 may provide a novel strategy for the development of targeted CMs proliferation drugs.

PHB complexes (PHBs) are the potent transcriptional co‐regulators encoding two proteins, PHB1 and PHB2, which are interdependent at the protein level, with deletion of one leading to simultaneous deletion of the other. PHBs are proteins with pleiotropic properties mediated in a cell‐compartment and tissue‐specific manner, including plasma membrane‐associated cell signaling functions, mitochondrial chaperones, and transcriptional co‐regulators of nuclear transcription factors, and are widely distributed in a variety of biological cells from different species. The functions of PHBs may be determined by their subcellular localization, with PHBs in the cell membrane regulating membrane‐transmitted cell signaling, PHBs in the nucleus controlling transcriptional activation and the cell cycle, and PHBs in mitochondria stabilizing the mitochondrial genome and regulating mitochondrial dynamics, mitochondrial morphology, mitochondrial biogenesis, and endogenous apoptosis pathways in mitochondria [52]. PHBs have different roles in different phases of the cell cycle. In the G_1_ phase, PHBs can prevent cells from entering the S phase of the cell cycle by inhibiting the activity of cyclin‐dependent kinase (CDK) until the cells are ready to enter S phase. In addition, PHBs are involved in the regulation of cell growth and metabolism to prepare cells for entry into S phase. During the S phase, PHBs may interact with DNA replication‐related proteins to ensure the accuracy and integrity of DNA replication. At the same time, PHBs may also regulate cellular metabolism and energy supply to support DNA replication. In G_2_/M phase, PHBs can work with checkpoint proteins to detect whether the cell is ready to enter mitosis and block cell cycle progression if necessary. Once the cell has passed the checkpoint, PHBs may also be involved in regulating the mitotic process to ensure correct chromosome segregation and normal cell division [53]. Compared with other phases of the cell cycle, the expression of PHBs increases approximately threefold upon entry into G_1_, which in turn can significantly promote the synthesis of RNA and related proteins [53]. Previous studies have shown that activation of the Ras‐induced Raf‐MEK‐ERK pathway is dependent on PHBs. Phosphorylated PHBs first bind to Raf to form a dimer, which is then recruited by GTP‐bound Ras proteins to form a multimer in the cell membrane, which in turn activates the Raf‐MEK‐ERK pathway [10, 11, 12, 13]. Subsequently, phosphorylated ERK translocates to the nucleus and initiates transcription of the cell cycle proteins CDK4, CDK6, CDC25, and Cyclin D, which promotes cell cycle re‐entry, enhances RNA and protein biosynthesis in G1 phase, and re‐initiates mitotic activity of cells, ultimately promoting cellular neogenesis. In addition, our results are consistent with previous studies showing that PHB1 knockdown results in the absence of PHB1 membrane localization and disruption of the PHB‐Raf complex, leading to cell cycle arrest in the G_0_/G_1_ phase and inactivation of the Raf‐MEK‐ERK pathway, resulting in the inability of cells to undergo mitosis [52]. This explains why overexpression of PHB1 can promote CMs proliferation and inhibition of PHB1 can inhibit CM re‐entry into the cell cycle, both in vivo and in vitro. Our current study showed that SalB interacts with PHBs and increases their phosphorylation with Raf.

CMs’ proliferation is a dynamic response of the heart to a variety of etiological stimuli such as ischemia‐reperfusion injury, HF, and valvular heart disease [54]. Interestingly, PHB1 knockdown reduced CMs mitosis and proliferation, suggesting that a pathological form of PHB1 upregulation may represent an organismal self‐protection against disease. Although the etiology of myocardial infarction varies, CMs infarction is the underlying pathophysiology that promotes the progression of HF. Therefore, targeting PHB1 would be a potentially effective approach to reverse myocardial infarction and the subsequent development of HF. Clinical studies have identified many factors, including age, fatty acids, exposure to pathogens, temperature regulation, and circadian rhythm, that influence the reversibility of myocardial infarction [5]. The therapeutic effects of drugs must be interpreted in the context of the underlying pathophysiology of myocardial infarction. Thus, the activity of SalB in the p‐MIHF‐induced myocardial infarction model may not necessarily apply to myocardial infarction caused by other etiologies, but warrants further investigation in the future.

In conclusion, we have provided a comprehensive characterization of the newly discovered PHB1 and provided new mechanistic insights into the role of PHB1 in the development of p‐MIHF‐induced CMs proliferation. We also provided compelling evidence that PHB1 is a bona fide target of a new TCM small molecule, SalB, which can effectively reverse infarct‐induced CMs proliferation by specifically binding to PHB1. When SalB binds to the C‐terminus of the PHB heterodimer, its binding mode with Raf undergoes a significant conformational change, enabling a closer binding between PHB1 and the Raf protein. This enhances the recognition and phosphorylation of Raf by Akt, thereby activating the Raf‐ERK signaling pathway and promoting the process of cell mitosis. Activation of PHB1 through small‐molecule targeting may be a novel and promising strategy not only to alleviate myocardial ischemia‐induced HF but also to promote myocardial infarction‐induced CMs proliferation.

## 4 Materials and Methods

### 4.1 Animals and Cells

We purchased 60 male SD rats, aged seven weeks and weighing between 180 and 200 g, from SPF (Beijing) biotechnology Co. Ltd. (Animal certificate number: SCXK (Beijing) 2019‐0010). The H9C2 cell line was obtained from the Cell Resource Center, Peking Union Medical College (which is part of the National Science and Technology Infrastructure, the National Biomedical Cell‐Line Resource, NSTI‐BMCR. http://cellresource.cn). The HCM was obtained from Beijing Cellapybio Co. Ltd. (cat. no. CA22001106, Beijing, China).

### 4.2 Cell Culture and Model

H9C2 cells were routinely digested and diluted to adjust the cell density to 5 × 10^4^ cells/mL and spread in the corresponding cell culture vessels. When the cell confluence reached more than 70 %, the cells were randomly divided into the following three groups: NC group, H/R group, and salvianolic acid B (25 µmol/L) group (H/R+SalB). The model group was given cell hypoxia for 9 h and reoxygenation for 3 h. H/R+SalB group was given 12 h of drug culture in advance based on the model. The dose of SalB was determined by reference to previous literature [55]. After 24 h of lentiviral co‐culture, H9C2 cells were randomly divided into four groups: overexpressed normal control group (OE‐NC), overexpressed PHB1 group (OE‐PHB1), short hairpin RNA group transfected with blank viral plasmids (sh‐NC), and short hairpin RNA group transfected with PHB1 viral plasmid (sh‐PHB1).

### 4.3 Apoptosis Analysis by Flow Detection

At the end of hypoxia‐reoxygenation, cells were digested routinely and washed twice with PBS. 100 µL of diluted dye reaction solution was added to each well, 5 µL of AnnexinV‐PE and 5 µL of 7‐AAD were added, respectively, and the reaction was performed at room temperature and protected from light for 30 min. 400 µL of PBS was replenished to each well, and the cells were resuspended and then detected by flow cytometry. CellQuest software was used to analyze the apoptosis data. AnnexinV‐PE was channel FL2, and 7‐AAD was channel FL4. FSC/SSC scatter plot circled the major cells, and AnnexinV/7‐AAD scatter plot was used to analyze the apoptosis.

### 4.4 Cell Cycle Analysis by Flow Cytometry

At the end of hypoxia‐reoxygenation, cells were routinely digested and washed twice with PBS. 1 mL of DNA staining solution and 10 µL of permeabilization solution were added, vortexed for 5–10 s, and mixed well, then incubated for 30 min at room temperature without light. The cell cycle was then detected by flow cytometry. The proportions of different cell cycles in each sample were analyzed using ModFit software.

### 4.5 Pull‐Down Assay

SalB was chemically coupled to magnetic nanoparticles as previously reported. SalB‐coupled beads (SalB beads) were mixed with H9C2 cell lysates and incubated for 2 h at 4°C. At the same time, uncoupled beads were used as a negative control. The bead‐captured proteins were then separated by 10% SDS–polyacrylamide gel electrophoresis and analyzed by Western blotting or Coomassie blue staining, followed by LC‐MS/MS analysis using a nano‐HPLC‐tandem LTO‐Orbitrap Velos pro mass spectrometer (Thermo Fisher Scientific).

### 4.6 Molecular Dynamics Simulation

The protein structures of PHB1, PHB2, and C‐Raf were obtained from the AlphaFold database (AF‐P35232, AF‐Q99623, and AF‐P04049, respectively) [56]. Molecular docking of SalB with the proteins was performed using AutoDock Vina [27, 28]. The heterodimeric complex structure of PHB1 and PHB2 was modeled using AlphaFold3 [57, 58]. Molecular dynamics (MD) simulations of the complexes were conducted using GROMACS [59, 60]. The system was solvated in a cubic box of TIP3P water molecules. To neutralize the system, chloride and sodium ions were randomly added to the simulation box. The CHARMM36 force field was employed for the simulations. The system was first energy‐minimized using the steepest descent algorithm, followed by a 500 ps equilibration in the canonical ensemble (NVT) and a 1000 ps equilibration in the isothermal‐isobaric ensemble (NPT). Finally, 100 ns MD simulations were performed for each complex. The MM/PBSA method was utilized to calculate the binding free energies of the proteins [61, 62]. Structural analysis and visualization were performed using GROMACS, VMD, and LigPlot+ [63, 64].

### 4.7 Biolayer Interferometry

The biosensor was equilibrated by immersing it in a buffer solution, and then immersed in a hardening solution containing PHB1. The biotinylated PHB1 in the solution binds to the surface of the biosensor, increasing the thickness of its surface film layer. The cured sensor was immersed in the baseline buffer solution. Next, the cured biosensor was immersed in the sample solution containing salvianolic acid B, which resulted in an increase in the thickness of the film layer due to the interspecific binding of PHB1 to salvianolic acid B. Finally, the sensor that has bound salvianolic acid B and PHB1 is dissociated by immersing it in a buffer solution, and salvianolic acid B is released from the surface of the biosensor, resulting in a decrease in the thickness of the membrane layer. The above data were monitored in real time and fed into the software for calculation.

### 4.8 Animal Experiments and Drug Administration

The experiment was divided into the sham operation group (Sham), post‐heart failure myocardial infarction group (Model), Model + salvianolic acid B (100 mg·kg^−1^) group (SalB), Model+ RNAi group (RNAi+ NC), Model+ RNAi+ PHB1 group (RNAi+ PHB1), and Model+ RNAi+ PHB1+ salvianolic acid B group (RNAi+ PHB1+ SalB). The dose of SalB was determined with reference to the previous literature [65]. BrdU was administered intraperitoneally at a concentration of 50 mg·kg^−1^. The establishment of the animal model was based on previous literature and improved according to the preliminary experiment and the previous experimental experience of the research group [66, 67].

### 4.9 Activity‐Based Protein Profiling

50.0 mg of protein, 30.4 mg of HATU, 6.9 mg of 3‐methyl‐3H‐bis‐acridine‐3‐ethanamine, and 12.9 mg of DIPEA were all dissolved in 10 mL of DMF and reacted overnight at room temperature, and the reaction process was monitored by TLC. At the end of the reaction, the solvent was spin‐dried, 1.5 mL of methanol was added, sonicated for 1–2 min, and DCM was added slowly dropwise to the solution while shaking until a large amount of white solid precipitated. Extraction was performed, and the filter cake was rinsed with PE to obtain the target compound. The small molecule and protein were separately premixed at a concentration of 0.5 µM and 0.1 nM, respectively, and incubated at 4°C for 1 h. Subsequently, UV fixation was performed for 15 min, after which 40 µL of sample loading buffer was added and boiled for 10 min. This was followed by SDS–polyacrylamide gel electrophoresis. The protein bands obtained were analyzed by ion chromatography–mass spectrometry. The mass spectrometry raw files were processed and converted by MM File Conversion software to obtain MGF format files, and then the UniProt database was searched using MASCOT (http://www.matrixscience.com/).

### 4.10 Statistics

Statistical analyses were performed using SPSS software program (version 26, Chicago, IL, USA). All data represent biological replicates (*n*) and were expressed as mean ± SD. Statistical significance was assessed using one‐way ANOVA with post hoc Student's *t*‐test. Differences were considered significant at ^*^ *p* < 0.05 or ^**^ *p* < 0.01.

Detailed experimental procedures, including materials, animal models, histological staining, echocardiography, and molecular biological assays, are provided in the Supporting Information Methods.

## Author Contributions

**Ce Cao, Bo Ma, and Jian Zhang**: writing – review and editing, writing – original draft, visualization, validation, supervision, formal analysis, conceptualization. **Lili Yang, Zixin Liu, Haoran Li, Jianshu Song, and Keer Zhang**: methodology, investigation. **Lei Li, Jianhua Fu**: writing – original draft, visualization, validation, supervision, funding acquisition, conceptualization. **Jianxun Liu**: writing – original draft, visualization, validation, supervision, funding acquisition, conceptualization. All authors have read and approved the final manuscript.

## Funding

This work was supported by the National Natural Science Foundation of China (no. 82574625, 82574858, and 82030124) and the Hospital capability enhancement project of Xiyuan Hospital, CACMS (no. XYZX0303‐03).

## Ethics Statement

Animal feeding and experimental operations were performed at the Experimental Animal Center of Xiyuan Hospital [SYXK (Beijing) 2023‐0053]. The treatment of experimental animals strictly followed the principles of “reduction, substitution, and optimization” and was provided humanely. All animal experiments complied with the ARRIVE guidelines. The Ethics Committee of Xiyuan Hospital approved the study (no. 2021XLC037, March 29 2021).

## Conflicts of Interest

The authors declare no conflicts of interest.

## Supporting Information

Additional supporting information may be found in the online version of the article at the publisher's website.

## Supporting information




**FIGURE S1**: Kaomas brilliant blue staining of binding protein. M is Marker,1 is total protein, 2 is the salvianolic acid B magnetic bead precipitate, 3 is the salvianolic acid B magnetic bead supernatant, and 4 is the BSA bead isolated protein.
**FIGURE S2**: Expression of PHB1 and its signaling pathway in heart failure patients was examined by the GEO database. (A) Schematic diagram of GEO experiments. (B) Expression of PHB1 and its signaling pathway in heart failure patients. Control, nonfailing donor; PPCM, peripartum cardiomyopathy; DCM, dilated cardiomyopathy; HCM, hypertrophic cardiomyopathy. Data are presented as the means ± SD compared with the control group, ^*^ *p* < 0.05, ^**^ *p* < 0.01.
**FIGURE S3**: Amino acids interacting with SalB in the structure of the PHB1‐SalB complex obtained by molecular docking.
**FIGURE S4**: RMSD of molecular dynamics simulations of PHB1 in complex with SalB.
**FIGURE S5**: Conformational changes during molecular dynamics simulations of PHB1 and SalB complexes. PHB1 is shown as a green cartoon representation, and SalB is depicted as an orange stick model.
**FIGURE S6**: SalB activates downstream pathways by altering the competitive phosphorylation of PHB1 with Raf. (A) Structure of the docking complexes of PHB1/2_Raf. (B) Structure of the docking complexes of PHB1/2_Raf_SalB. (C) RMSD of molecular dynamics simulations of PHB1/2 in complex with Raf. (D) RMSD of molecular dynamics simulations of PHB1/2_SalB complexed with Raf. PHB1, PHB2, and Raf are shown in green, blue, and purple cartoon representations, respectively, while SalB is depicted as an orange stick model.
**FIGURE S7**: Conformational changes during molecular dynamics simulations of PHB1/2 complexes with Raf. PHB1, PHB2, and Raf are shown in green, blue, and purple cartoon representations, respectively.
**FIGURE S8**: Conformational changes during molecular dynamics simulations of PHB1/2_SalB complexes with Raf. PHB1, PHB2, and Raf are shown in green, blue, and purple cartoon representations, respectively, while SalB is depicted as an orange stick model.
**FIGURE S9**: SalB altered the binding sites of PHB1/2 and Raf. PHB1, PHB2, and Raf are shown in green, blue, and purple surface representations, respectively.
**FIGURE S10**: Conformational change of PHB1/2 C‐terminus induced by SalB. (A) Structural alignment of the SalB‐bound and unbound PHB1/2 complexes based on the C‐terminal helix. (B) SalB‐unbound PHB1/2. (C) SalB‐bound PHB1/2. The SalB‐unbound PHB1/2 is shown as a yellow cartoon representation, the SalB‐bound PHB1/2 is shown as a green cartoon representation, and SalB is depicted as a green stick model.
**FIGURE S11**: SalB Binding Modulates the C‐Terminal Conformation of PHB1/2 and Enhances Its Interaction with Raf. (A) Hydrogen bonds in the C‐terminal structure of SalB‐unbound PHB1/2. (B) Hydrogen bonds in the C‐terminal structure of SalB‐bound PHB1/2. (C) Interaction between SalB and the C‐terminal region of PHB1/2. SalB is shown in pink stick representation. (D) Hydrogen bonding between the C‐terminus of SalB‐unbound PHB1/2 and Raf. (E, F) Hydrogen bonding between the C‐terminus of SalB‐bound PHB1/2 and Raf. SalB is depicted as a green stick model. In all panels, the SalB‐unbound PHB1/2 is illustrated as a yellow cartoon, the SalB‐bound PHB1/2 as a green cartoon, and Raf in a purple cartoon representation. Residues involved in hydrogen bonding and hydrophobic interactions are displayed as sticks. Hydrogen bonds are indicated by black dashed lines, and hydrophobic interactions are shown as solid yellow lines.
**FIGURE S12**: Western blot analysis of PHB1, p‑PHB1, p‑A‑Raf, A‑Raf, p‑C‑Raf, C‑Raf, p‑ERK1/2, ERK1/2, CDK4, Cyclin D1 and GAPDH in OE‑NC, OE‑PHB1, sh‑NC, and sh‑PHB1 groups (sample 1 and 2). Each lane corresponds to the indicated group in order.
**FIGURE S13**: Western blot analysis of PHB1, p‑PHB1, p‑A‑Raf, A‑Raf, p‑C‑Raf, C‑Raf, p‑ERK1/2, ERK1/2, CDK4, CDK6, Cyclin D1, CDC25 and GAPDH in OE‑NC, OE‑PHB1, sh‑NC, and sh‑PHB1 groups (sample 3 and 4). Each lane corresponds to the indicated group in order.
**FIGURE S14**: Western blot analysis of PHB1, p‑PHB1, p‑A‑Raf, A‑Raf, p‑C‑Raf, C‑Raf, p‑ERK1/2, ERK1/2, CDK4, CDK6, Cyclin D1, CDC25 and GAPDH in NC, H/R, and H/R+SalB groups. Each lane corresponds to the indicated group in order.
**FIGURE S15**: Western blot analysis of PHB1, p‑PHB1, p‑A‑Raf, A‑Raf, p‑C‑Raf, C‑Raf, p‑ERK1/2, ERK1/2, CDK4, CDK6, Cyclin D1, CDC25 and GAPDH in Sham, Model, SalB, RNAi+NC, RNAi+PHB1, and RNAi+PHB1+SalB groups. Each lane corresponds to the indicated group in order.
**FIGURE S16**: Expression levels of PHB1, p‑PHB1, p‑A‑Raf, A‑Raf, p‑C‑Raf, C‑Raf, p‑ERK1/2, ERK1/2, CDK4, CDK6, and Cyclin D1 in human cardiomyocytes with myocardial infarction‑related heart failure (*n* = 3). Each lane corresponds to NC, H/R, H/R+SalB, H/R+LV‑NC, H/R+LV‑PHB1, and H/R+LV‑PHB1+SalB groups in order.
**FIGURE S17**: Western blot analysis of PHB1, p‑PHB1, p‑A‑Raf, A‑Raf, p‑C‑Raf, p‑ERK1/2, ERK1/2, CDK4, CDK6, Cyclin D1, and GAPDH in human cardiomyocytes. Each lane corresponds to NC, H/R, H/R+SalB, H/R+LV‑NC, H/R+LV‑PHB1, and H/R+LV‑PHB1+SalB groups in order.

## Data Availability

The datasets used and/or analyzed during the present study are available from the corresponding author on reasonable request.
