# Supplementary material for: A Novel Mechanism of Salvianolic Acid B in Postmyocardial Infarction Cardiac Protection: PHB1‐Driven Raf‐ERK Pathway Activation Promotes Cardiomyocyte Mitosis
Source: MedComm (2020). 2026 May 3;7(5):e70752. doi: 10.1002/mco2.70752 (PMC13136069; doi:10.1002/mco2.70752)
Supplement: Supplementary file 1 — FIGURE S1: Kaomas brilliant blue staining of binding protein. M is Marker,1 is total protein, 2 is the salvianolic acid B magnetic bead precipitate, 3 is the salvianolic acid B magnetic bead supernatant, and 4 is the BSA bead isolated protein. FIGURE S2: Expression of PHB1 and its signaling pathway in heart failure patients was examined by the GEO database. (A) Schematic diagram of GEO experiments. (B) Expression of PHB1 and its signaling pathway in heart failure patients. Control, nonfailing donor; PPCM, peripartum cardiomyopathy; DCM, dilated cardiomyopathy; HCM, hypertrophic cardiomyopathy. Data are presented as the means ± SD compared with the control group, * p < 0.05, ** p < 0.01. FIGURE S3: Amino acids interacting with SalB in the structure of the PHB1‐SalB complex obtained by molecular docking. FIGURE S4: RMSD of molecular dynamics simulations of PHB1 in complex with SalB. FIGURE S5: Conformational changes during molecular dynamics simulations of PHB1 and SalB complexes. PHB1 is shown as a green cartoon representation, and SalB is depicted as an orange stick model. FIGURE S6: SalB activates downstream pathways by altering the competitive phosphorylation of PHB1 with Raf. (A) Structure of the docking complexes of PHB1/2_Raf. (B) Structure of the docking complexes of PHB1/2_Raf_SalB. (C) RMSD of molecular dynamics simulations of PHB1/2 in complex with Raf. (D) RMSD of molecular dynamics simulations of PHB1/2_SalB complexed with Raf. PHB1, PHB2, and Raf are shown in green, blue, and purple cartoon representations, respectively, while SalB is depicted as an orange stick model. FIGURE S7: Conformational changes during molecular dynamics simulations of PHB1/2 complexes with Raf. PHB1, PHB2, and Raf are shown in green, blue, and purple cartoon representations, respectively. FIGURE S8: Conformational changes during molecular dynamics simulations of PHB1/2_SalB complexes with Raf. PHB1, PHB2, and Raf are shown in green, blue, and purple cartoon representations, respe [file MCO2-7-e70752-s001.docx]

Supplementary Materials for

**A Novel Mechanism of Salvianolic Acid B in Post-Myocardial Infarction Cardiac Protection: PHB1-Driven Raf-ERK Pathway Activation Promotes Cardiomyocyte Mitosis**

Ce Cao *et al.*

**This PDF file includes:**

Supplementary Methods 1 to 9

FIGURE S1 to S17

Supplementary Methods

1 | Materials

The Salvianolic acid B was produced from Chengdu Desite Biotechnology Co. (Cat. no. DD0468, 97 % purity, Chengdu, China) and National Institutes for Food and Drug Control (Cat. no. 214P-LGZC, Beijing, China). Rat ANG2 ELESA Plate, Rat BNP ELESA Plate and Rat ANP ELESA Plate were obtained from Elabscience Biotechnology Co., Ltd. (Cat. no. E-EL-R0125, E-EL-R0126, E-EL-R0017, Wuhan, China). Creatine Kinase and Lactate Dehydrogenase were obtained from Rayto Life and Analytical Sciences Co., Ltd. (Cat. no. S03024, S03034, Shenzhen, China). Creatine Kinase, MB Form was obtained from Changchun Huili Biotechnology Co. (Cat. no.C060, Changchun, China). BrdU was obtained from Beyotime Biotech Inc. (Cat. no. ST1056-2g, Shanghai, China). Lentivirus, for in vitro cellular knockdown and overexpression of the PHB1 gene, and PHB1 adeno-associated virus, for in vivo knockdown of the PHB1 gene in SD rats, were purchased from Shanghai GeneChem Co., Ltd. (Lot. no. GCPL0410894, GCPL0410895, GIDV0390203, Shanghai, China). The Recombinant Anti- PHB1 antibody (Cat. no. ab75766), Anti-Cdk4 antibody (Cat. no. ab199728) and Anti-Cyclin D1 antibody (Cat. no. ab134175) were contained from Abcam Plc (Cambridge, United Kingdom). Phospho-A-Raf (Tyr302) Polyclonal Antibody (Cat. no.PA5-36783), Phospho-PHB1 (Thr258) (Cat. no PA5-37681) and Cdc25A Polyclonal Antibody (Cat. no. PA5-109500) were contained from Thermo Fisher Scientific Inc. (MA, United States). A-Raf Antibody (Cat. no. 4432), Phospho-c-Raf Antibody (Cat no. 9421), c-Raf Antibody (Cat. no. 53745), Phospho-p44/42 MAPK (Erk1/2) Antibody (Cat. no. 4370), p44/42 MAPK (Erk1/2) Antibody (Cat. no. 4695) and CDK6 Antibody (Cat. no .3136) were purchased from Cell Signaling Technology, Inc. (MA, United States). Recombinant Human PHB1 was obtained from CUSABIO Technology LLC (Houston, United States). The Dynabeads M-270 Amine (Cat. no.14308D) was obtained from Thermo Fisher Scientific Inc. (MA, United States). Phospho-Histone H3 (Ser10) Monoclonal antibody (Cat. no. 66863-1-Ig) and Nkx2.5 Polyclonal antibody (Cat. no. 13921-1-AP) were purchased from Proteintech Group, Inc. (Wuhan, China)

2 | Experimental instruments

The instruments included an ALC-V8 two-channel small-animal artificial respiration system (Jingyi Nuotai Biological Development Co., Ltd.), a Vevo2100 high-resolution ultrasound imaging system (VisualSonics Company of Canada), a microtome (Shanghai Leica Instrument Co., Ltd.), a D90 digital single-mirror reflex camera (Nikon Company of Japan), a CE-01 small-animal electrocardiograph (Beijing Ruanlong Biotechnology Co., Ltd.), an MP-150 refrigerated centrifuge (Thermo Fisher Scientific, USA), an MP-150 polygraph (BioPac, USA), an RM22% paraffin microtome, an EG1150C paraffin embedding apparatus (Leica, Germany), a Leica CM1860 cryostat (Leica Microsystems Inc.), a Bruker Daltonics ImagePrep electronic matrix sprayer (Bremen, Germany), an Epson Perfection V550 photo scanner (Epson Inc., Suwa, Japan) and an Octet BLI (Sartorius AG, Germany).

3 | Animal experiments

The room was maintained at a constant temperature of 26 °C, relative humidity of 70 %, and a 12 h light/12 h dark cycle. Wistar rats were anesthetized with 1% sodium pentobarbital (50 mg·kg^-1^) by intraperitoneal anesthesia, and the small-animal ventilator for artificial respiration and the small-animal electrocardiograph were connected. Anesthesia was complete when muscle tension, corneal reflexes, and response to skin pinching disappeared in the rats. The chest was then opened and the pericardium was exposed by breaking the 3-4 ribs. When the heart was exposed, a suture was inserted approximately 2 mm below the bifurcation of the left anterior descending coronary artery. Tightening the silk thread to cause myocardial ischemia, meanwhile, the judgment criteria for ischemia success were adopted by using the electrocardiogram QRS amplitude to increase, ST-segment elevation and T-wave towering or inversion. Notably, the sham operation group only underwent threading without ligation, and other operations were the same as the model group. After the experiment, the carcasses of the experimental animals were properly disposed according to the relevant national regulations. All animal feeding methods and experimental operations were approved by the medical ethics committees of Xiyuan Hospital, Chinese Academy of Chinese Medical Sciences. The virus group was injected by tail vein at the same time as the model was established. Salvianolic acid B was administered by gavage 24 h after operation for 2 weeks.

4 | Pathological changes of myocardial tissue detected by Hematoxylin and Eosin staining

Rats were anesthetized by intraperitoneal injection of pentobarbital sodium. At the same time, the heart was quickly removed, blotted dry with filter paper, and placed in a -20°C refrigerator for quick freezing for approximately 10 to 15 min. 5 uniform slices of approximately 1 mm thickness were cut from the heart, and then the slices were quickly placed in a 6-well plate containing TTC staining solution and incubated at 37 °C for 15-30 min in the dark. After the color development of the section was completed, the slices were placed in a special solution in 10 % formalin for some time to enhance the contrast. The stained tissue was then removed and rinsed with normal saline to remove excess staining solution from the surface of the tissue. Image J was used to measure and analyze infarct size and total myocardial area (calculation formula: percentage of infarct area = total infarct area/total myocardial area × 100 %).

5 | Echocardiography

Rats were anesthetized by intraperitoneal injection of pentobarbital sodium. Extensive skin preparation was performed on the left anterior thoracic region and the animals were restrained in the supine position. When the heart rhythm became regular, recording began. A high-frequency probe was used for positioning, and M-mode echocardiography was used to observe cardiac motion in each section of the left ventricular long axis and parasternal short axis. Left ventricular end-diastolic internal diameter, left ventricular end-systolic internal diameter, left ventricular end-diastolic volume, left ventricular end-systolic volume, and left ventricular ejection fraction were calculated from the left ventricular long-axis view, and we selected the average values of five consecutive cardiac cycles for each index.

6 | Rat myocardial tissue Masson staining

After echocardiography was completed, the heart was cut along the measured left rib of the rat and removed. After rinsing well with saline, the heart was placed on an anvil and sectioned 1/3 of the way down the ligature line. The middle section was fixed in 4 % paraformaldehyde solution, and paraffin sections were deparaffinized in water and then stained. Microscopic examination and image acquisition were performed under a microscope, and data analysis of the images was performed using Image J.

7 | Detection of serum indicators

Rats were anesthetized by intraperitoneal injection of pentobarbital sodium. Blood was collected from the abdominal aorta, allowed to stand at room temperature for 1 h, and centrifuged at 3500 rpm for 10 min to separate the serum. After the serum sample was collected and the reagents were prepared according to the instructions of the CK, CK-MB, LDH, ANP, BNP, and ANG2 kits, and the reagents were mixed proportionally with animal serum samples, the levels of CK, CK-MB, LDH, ANP, BNP, and ANG2 were calculated using a microplate reader.

8 | Rat myocardial tissue immunofluorescence

After echocardiography was completed, the heart was cut along the left thorax of the rats, removed, rinsed with saline, placed on an anvil, and fixed in 4 % paraformaldehyde solution after sectioning the lower 1/3 of the ligature. After paraffin sectioning, the sections were sequentially placed in dewaxing solution Ⅰ for 10 min, environmentally safe dewaxing solution Ⅱ for 10 min, environmentally safe dewaxing solution Ⅲ for 10 min, anhydrous ethanol Ⅰ for 5 min, anhydrous ethanol Ⅱ for 5 min, anhydrous ethanol Ⅲ for 5 min, and distilled water for washing. The slides were placed on a shaker and washed 3 times. After the slides were dried, BSA was added dropwise and sealed for 30 min, and the slides were incubated overnight at 4 ℃ in a wet box after the addition of primary antibody. After the primary antibody was removed, the slides were washed three times with PBS (pH 7.4), and then the corresponding secondary antibody was added and incubated for 50 min at room temperature without light. DAPI staining solution was added dropwise and incubated for 10 min at room temperature without light. After 3 washes, spontaneous fluorescence quencher was added for 5 min and rinsed with running water for 10 min. Images were captured and analyzed using Image J after the addition of the anti-fluorescence quenching sealer.

9 | Western blot assays

To obtain protein from rat myocardial tissue, rats were anesthetized by intraperitoneal injection of sodium pentobarbital. The ischemic and peripheral myocardial tissues were collected under ligature and cut into pieces with surgical scissors. Tissue cells were lysed with RIPA lysis buffer (containing protease and phosphatase inhibitors). To obtain the protein of the cells, a certain amount of PBS was added to the cell pellet and mixed, after the cells were suspended in PBS, the cell suspension was transferred to a glass homogenization tube in the ice-water mixture. After manual homogenization for 3 min, the broken cell suspension can be used for measurement. The protein was transferred to the PVDF membrane by determining the concentration using the BCA protein detection kit. The membranes were incubated with primary antibodies against p-PHB1, PHB1, p-A-Raf, A-Raf, p-C-Raf, C-Raf, p-ERK1/2, ERK1/2, CDK4, CDK6, CDC25A, Cyclin D1 and β-actin overnight at 4°C. The membranes were then washed with TBST buffer and incubated with secondary antibodies in TBST buffer for 1 h at 37 °C. Finally, the membranes were washed and visualized with Super ECL Western Blotting Substrate, and the bands were scanned and quantified using the ImageJ system

FIGURE S1


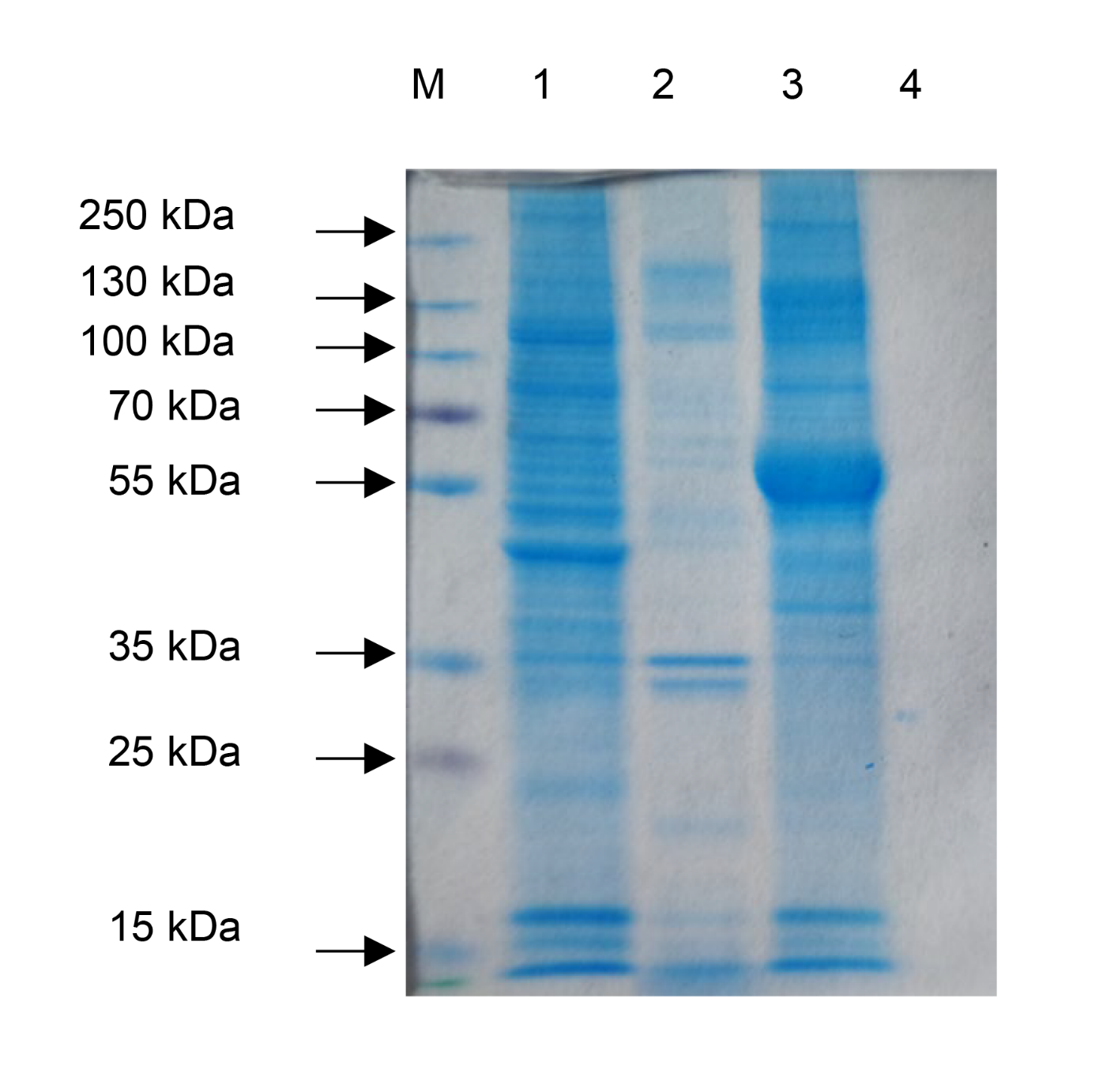


**FIGURE S1** Kaomas brilliant blue staining of binding protein. M is Marker,1 is total protein, 2 is the Salvianolic acid B magnetic bead precipitate, 3 is the Salvianolic acid B magnetic bead supernatant, 4 is BSA bead isolated protein.

**FIGURE S2**


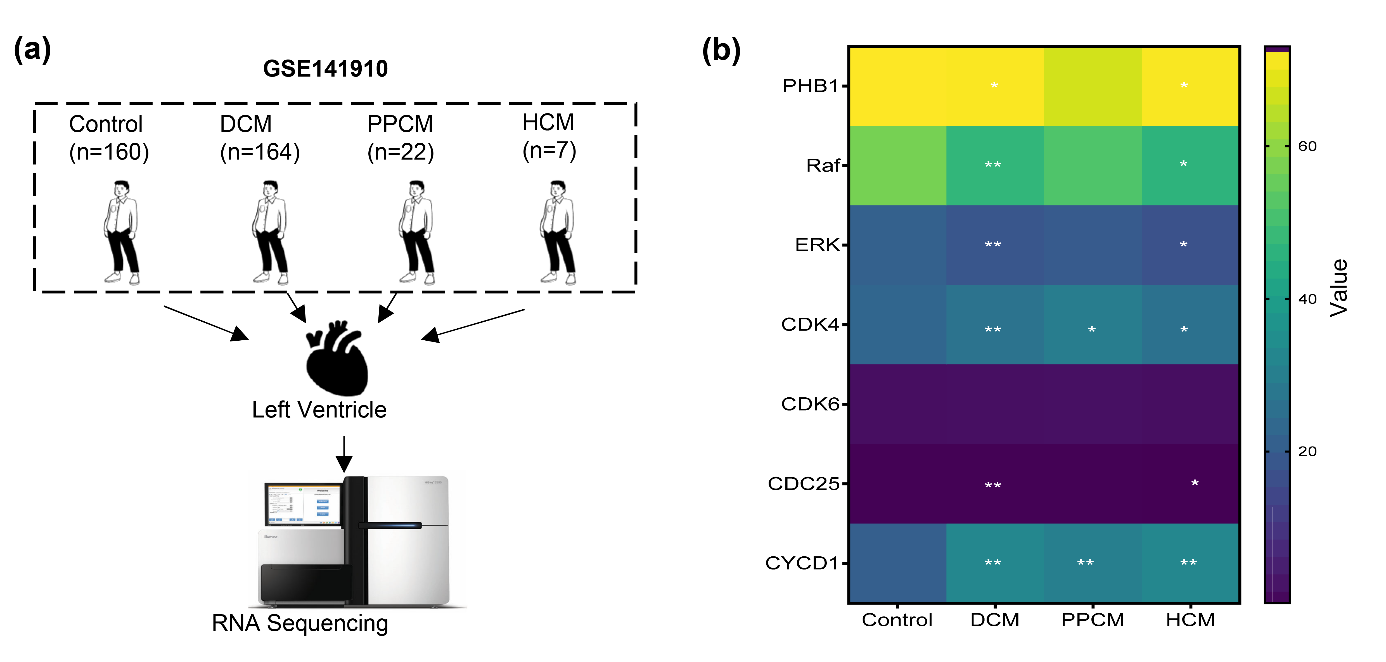


**FIGURE S2** Expression of PHB1 and its signaling pathway in heart failure patients was examined by the GEO database. (A), Schematic diagram of GEO experiments. (B), Expression of PHB1 and its signaling pathway in heart failure patients. Control, Non-Failing Donor; PPCM, Peripartum cardiomyopathy; DCM, Dilated cardiomyopathy; HCM, Hypertrophic cardiomyopathy. Data are presented as the means ± SD. compared with control group, ^*^*p*<0.05, ^**^*p*<0.01.

**FIGURE S3**


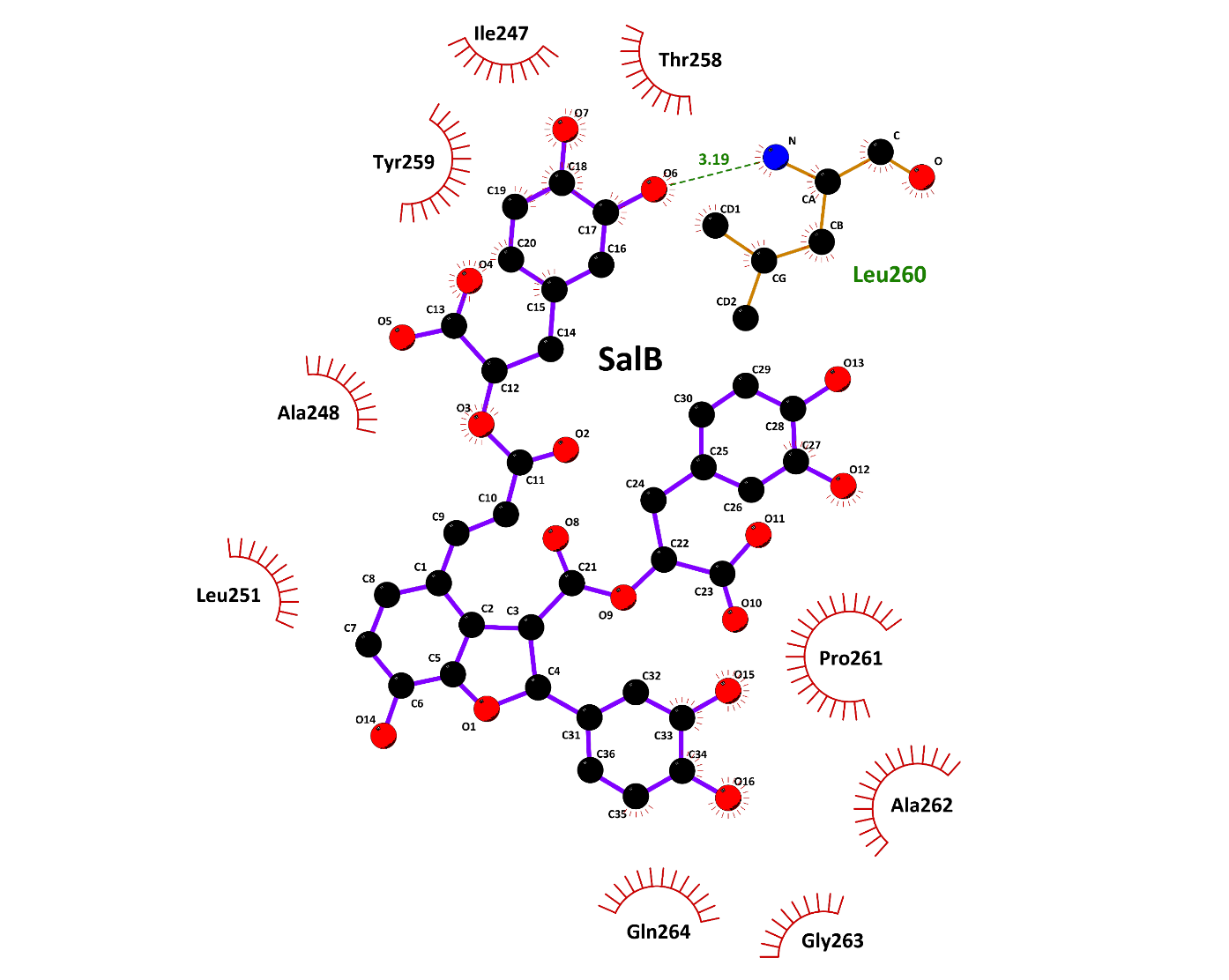


**FIGURE S3** Amino acids interacting with SalB in the structure of the PHB1-SalB complex obtained by molecular docking.

**FIGURE S4**


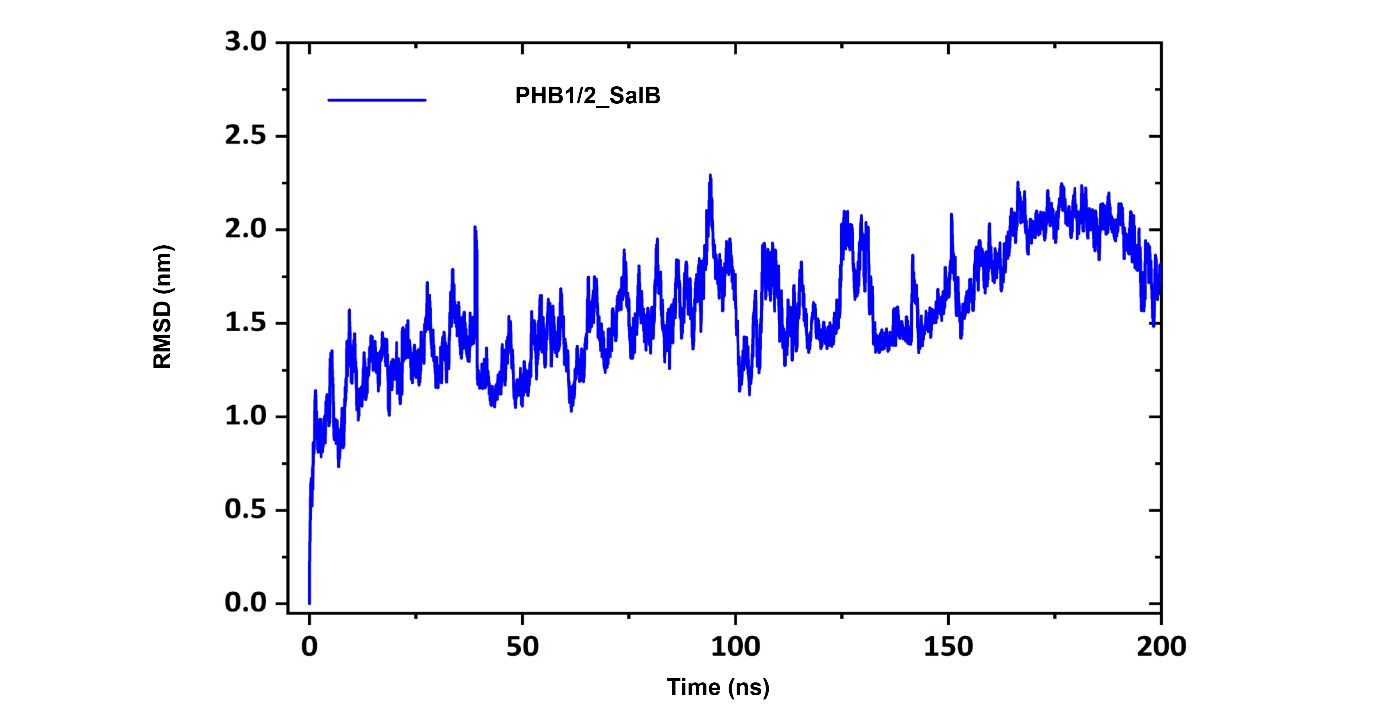


**FIGURE S4** RMSD of molecular dynamics simulations of PHB1 in complex with SalB.

**FIGURE S5**


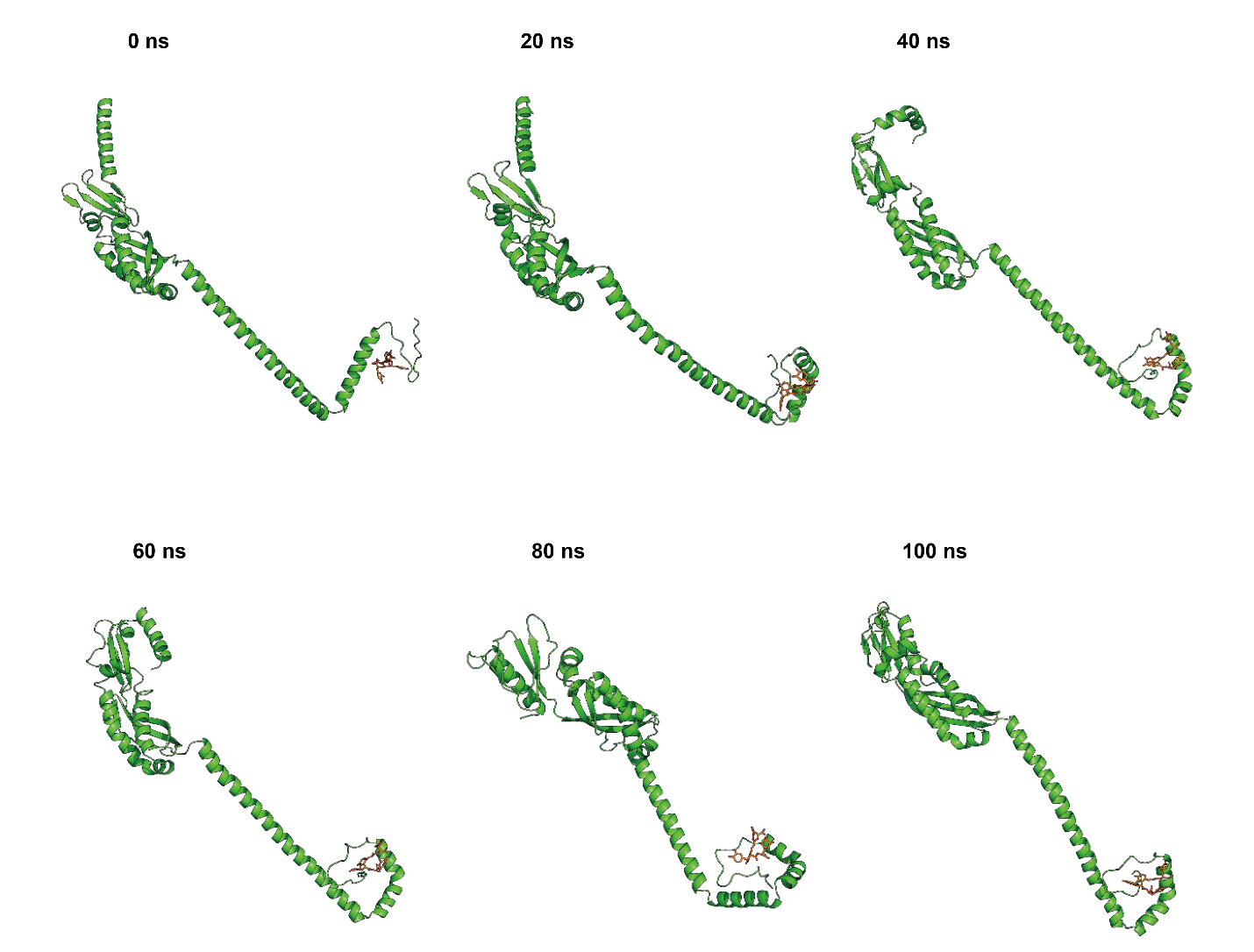


**FIGURE S5** Conformational changes during molecular dynamics simulations of PHB1 and SalB complexes. PHB1 is shown as a green cartoon representation, and SalB is depicted as a orange stick model.

**FIGURE S6**

**
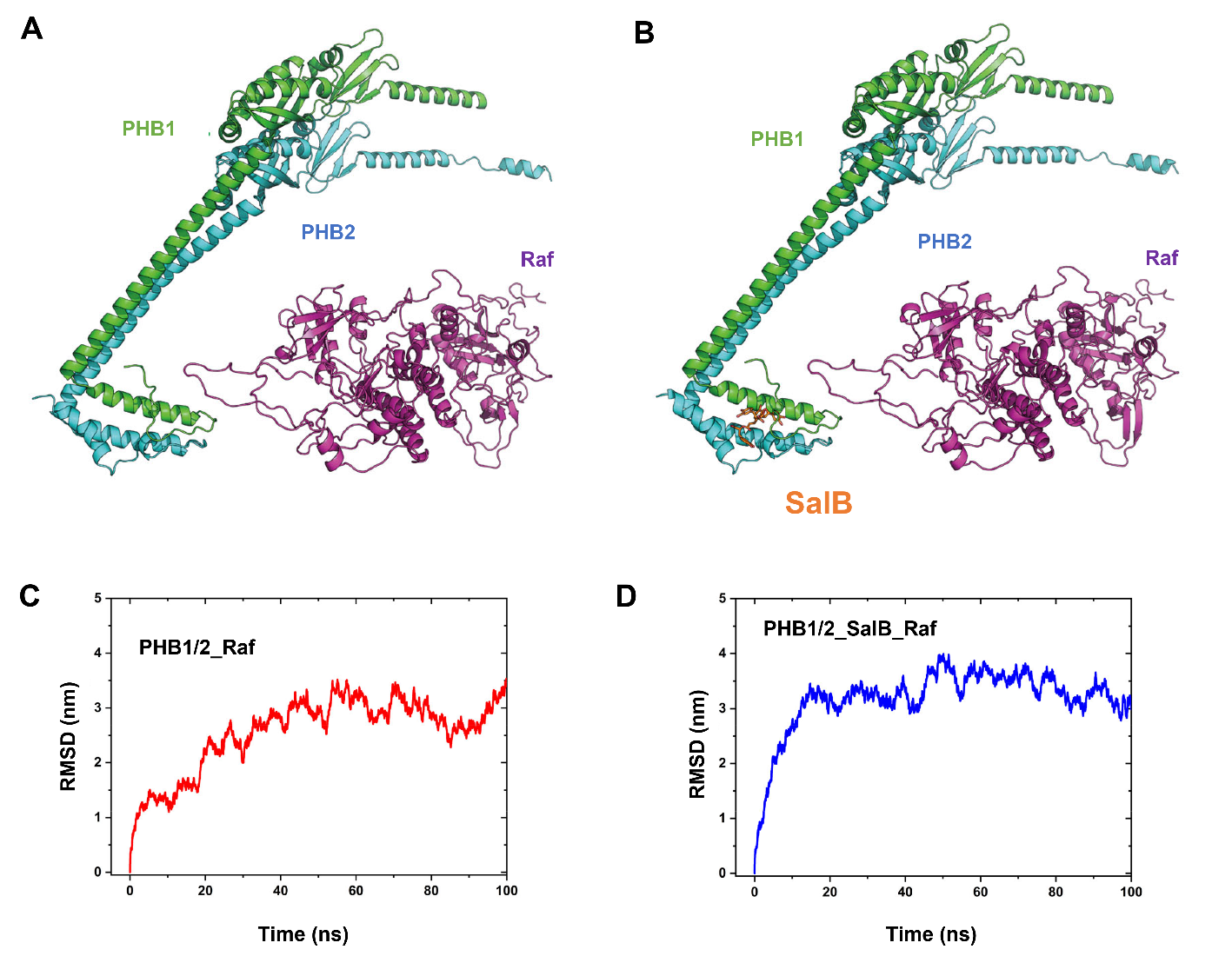
**

**FIGURE S6** SalB activates downstream pathways by altering the competitive phosphorylation of PHB1 with Raf. (a) Structure of the docking complexes of PHB1/2_Raf. (b) Structure of the docking complexes of PHB1/2_Raf_SalB. (c) RMSD of molecular dynamics simulations of PHB1/2 in complex with Raf. (d) RMSD of molecular dynamics simulations of PHB1/2_SalB complexed with Raf. PHB1, PHB2, and Raf are shown in green, blue, and purple cartoon representations, respectively, while SalB is depicted as an orange stick model

**FIGURE S7**

**
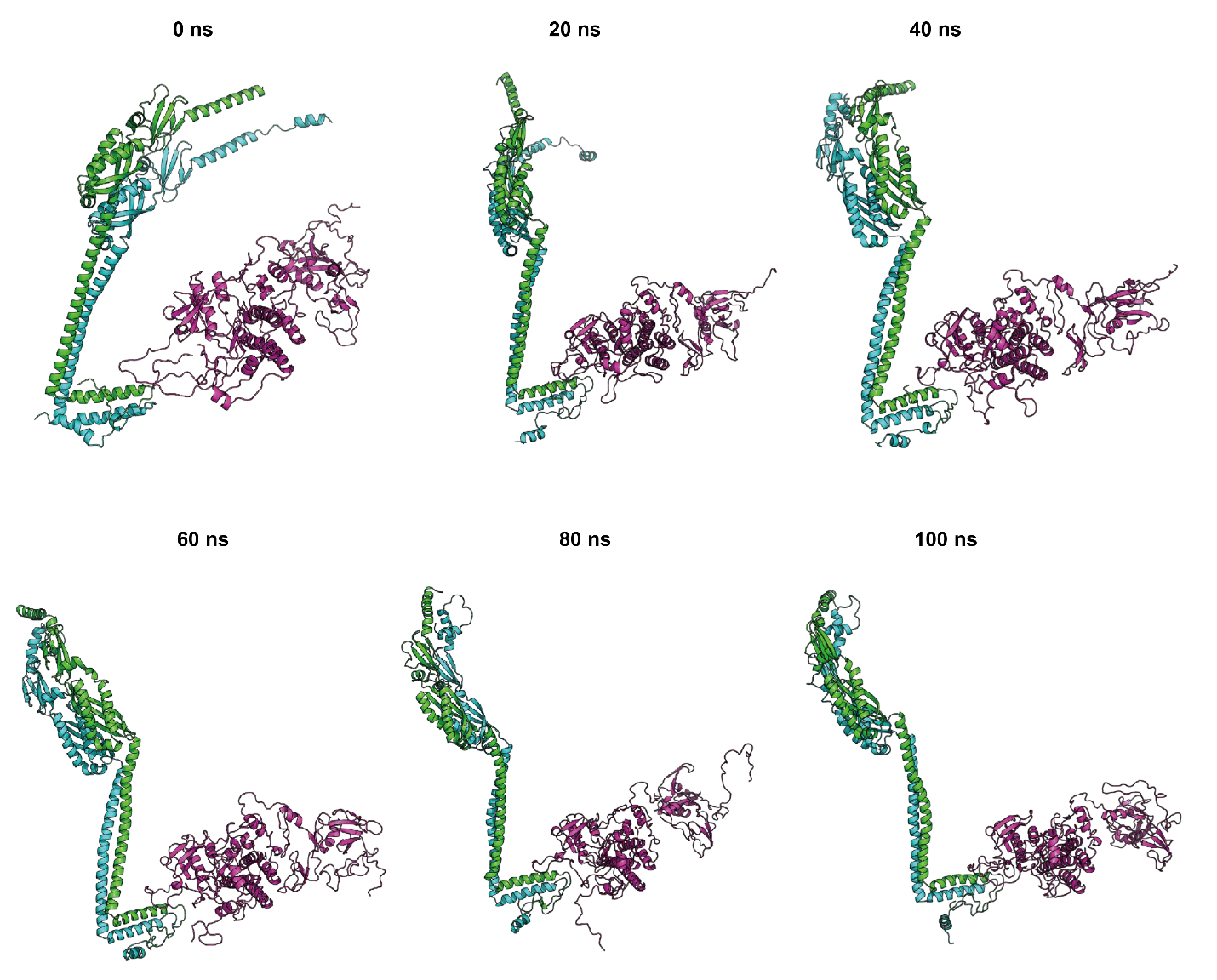
**

**FIGURE S7** Conformational changes during molecular dynamics simulations of PHB1/2 complexes with Raf. PHB1, PHB2, and Raf are shown in green, blue, and purple cartoon representations, respectively.

**FIGURE S8**


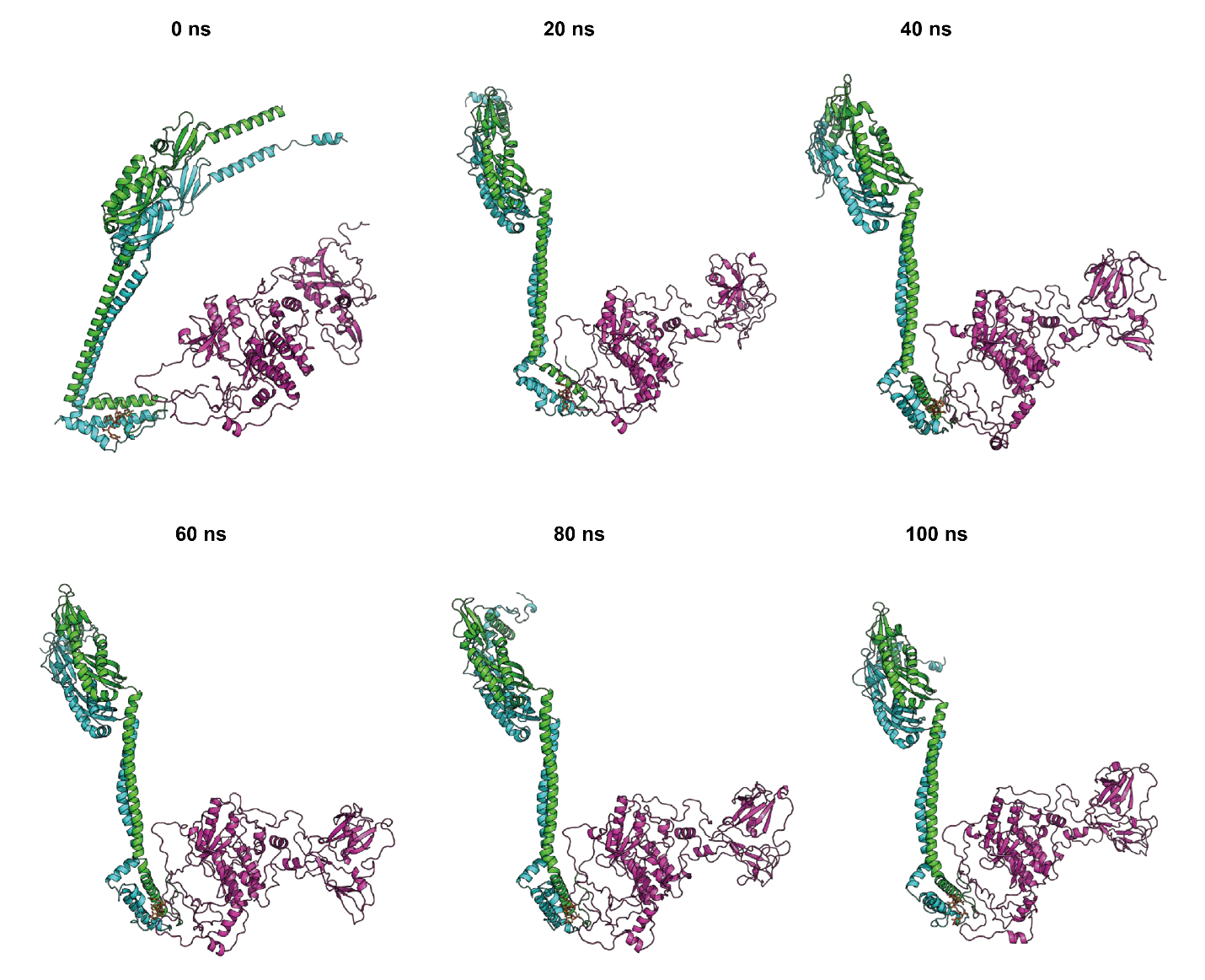


**FIGURE S8** Conformational changes during molecular dynamics simulations of PHB1/2_SalB complexes with Raf. PHB1, PHB2, and Raf are shown in green, blue, and purple cartoon representations, respectively, while SalB is depicted as an orange stick model.

**FIGURE S9**


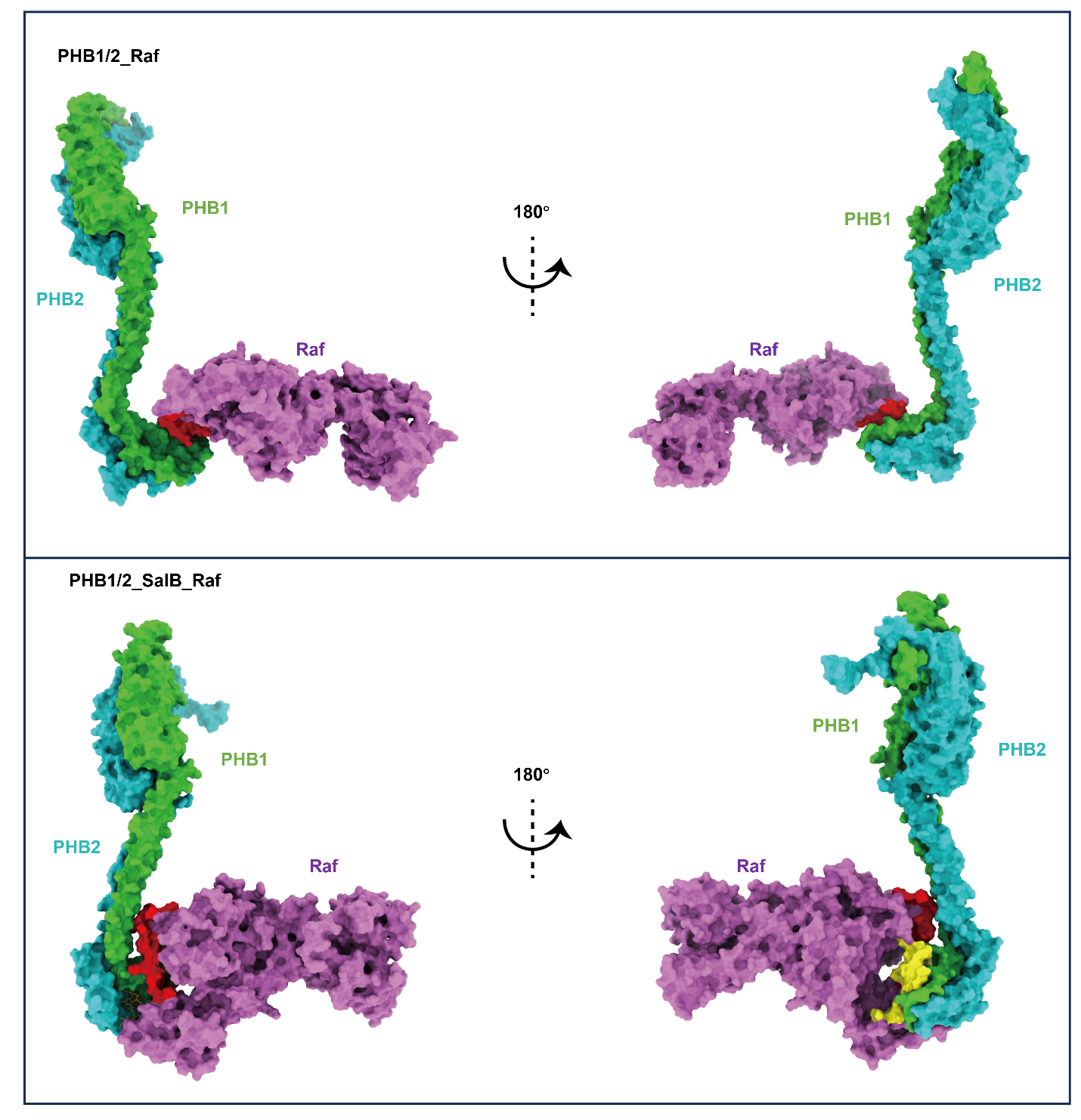


**FIGURE S9** SalB altered the binding sites of PHB1/2 and Raf. PHB1, PHB2, and Raf are shown in green, blue, and purple surface representations, respectively.

**FIGURE S10**

**
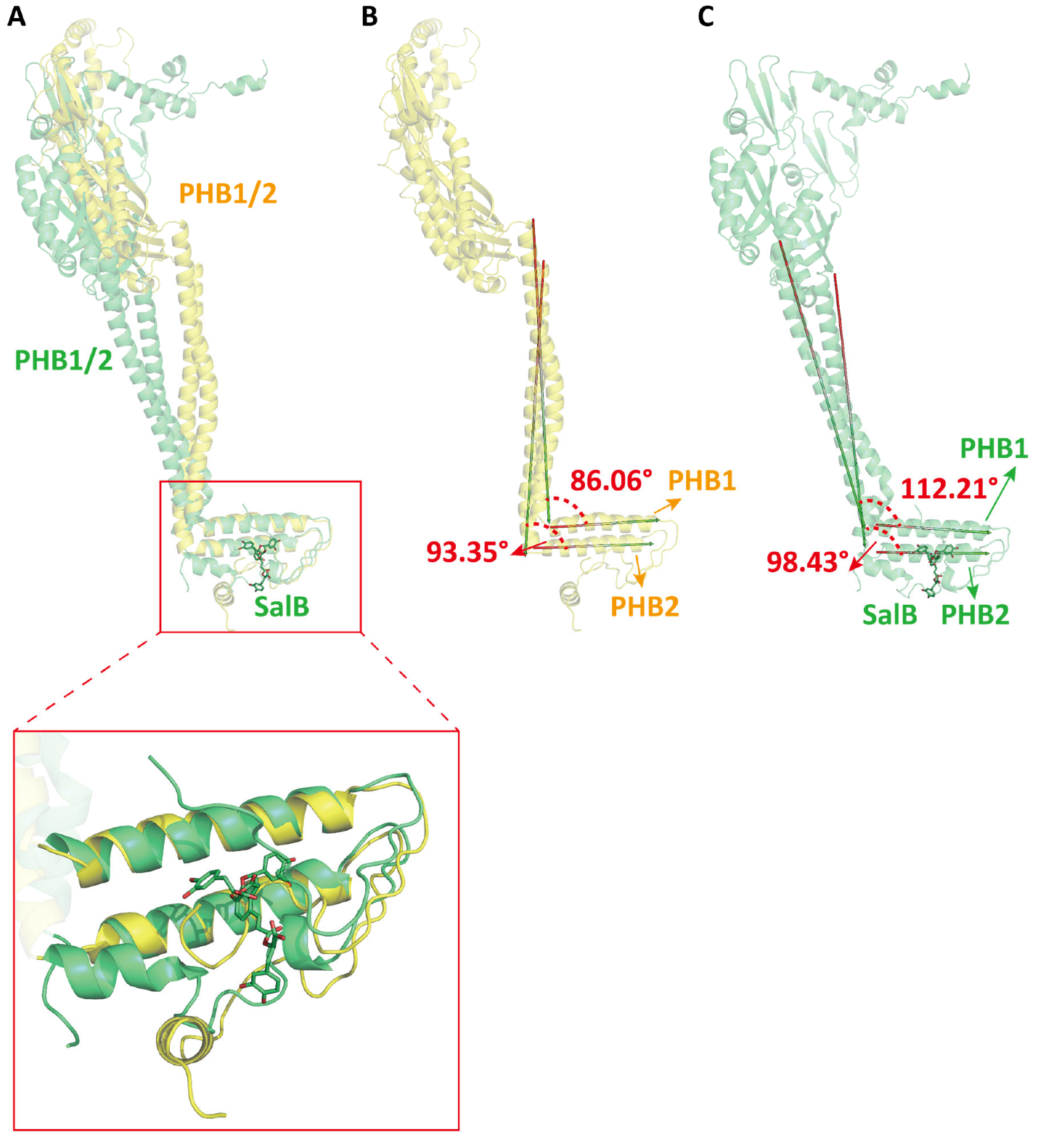
**

**FIGURE S10** Conformational change of PHB1/2 C-terminus induced by SalB. (A) Structural alignment of the SalB-bound and unbound PHB1/2 complexes based on the C-terminal helix. (B) SalB-unbound PHB1/2. (C) SalB-bound PHB1/2. The SalB-unbound PHB1/2 is shown as a yellow cartoon representation, the SalB-bound PHB1/2 is shown as a green cartoon representation, and SalB is depicted as a green stick model.

**FIGURE S11**

**
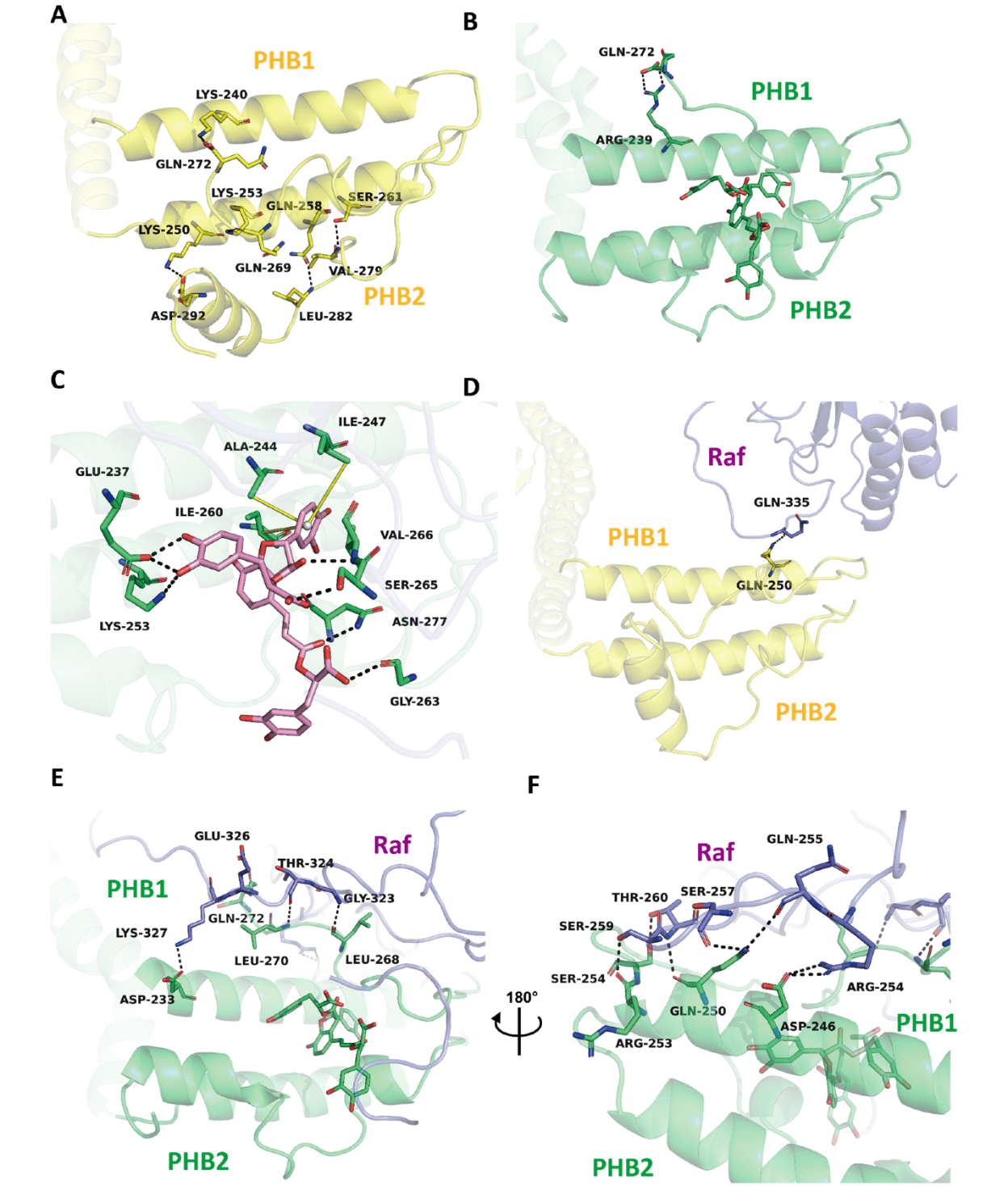
**

**FIGURE S11** SalB Binding Modulates the C-Terminal Conformation of PHB1/2 and Enhances Its Interaction with Raf. (A) Hydrogen bonds in the C-terminal structure of SalB-unbound PHB1/2. (B) Hydrogen bonds in the C-terminal structure of SalB-bound PHB1/2. (C) Interaction between SalB and the C-terminal region of PHB1/2. SalB is shown in pink stick representation. (D) Hydrogen bonding between the C-terminus of SalB-unbound PHB1/2 and Raf. (E, F) Hydrogen bonding between the C-terminus of SalB-bound PHB1/2 and Raf. SalB is depicted as a green stick model. In all panels, the SalB-unbound PHB1/2 is illustrated as a yellow cartoon, the SalB-bound PHB1/2 as a green cartoon, and Raf in purple cartoon representation. Residues involved in hydrogen bonding and hydrophobic interactions are displayed as sticks. Hydrogen bonds are indicated by black dashed lines, and hydrophobic interactions are shown as solid yellow lines.

**FIGURE S12**


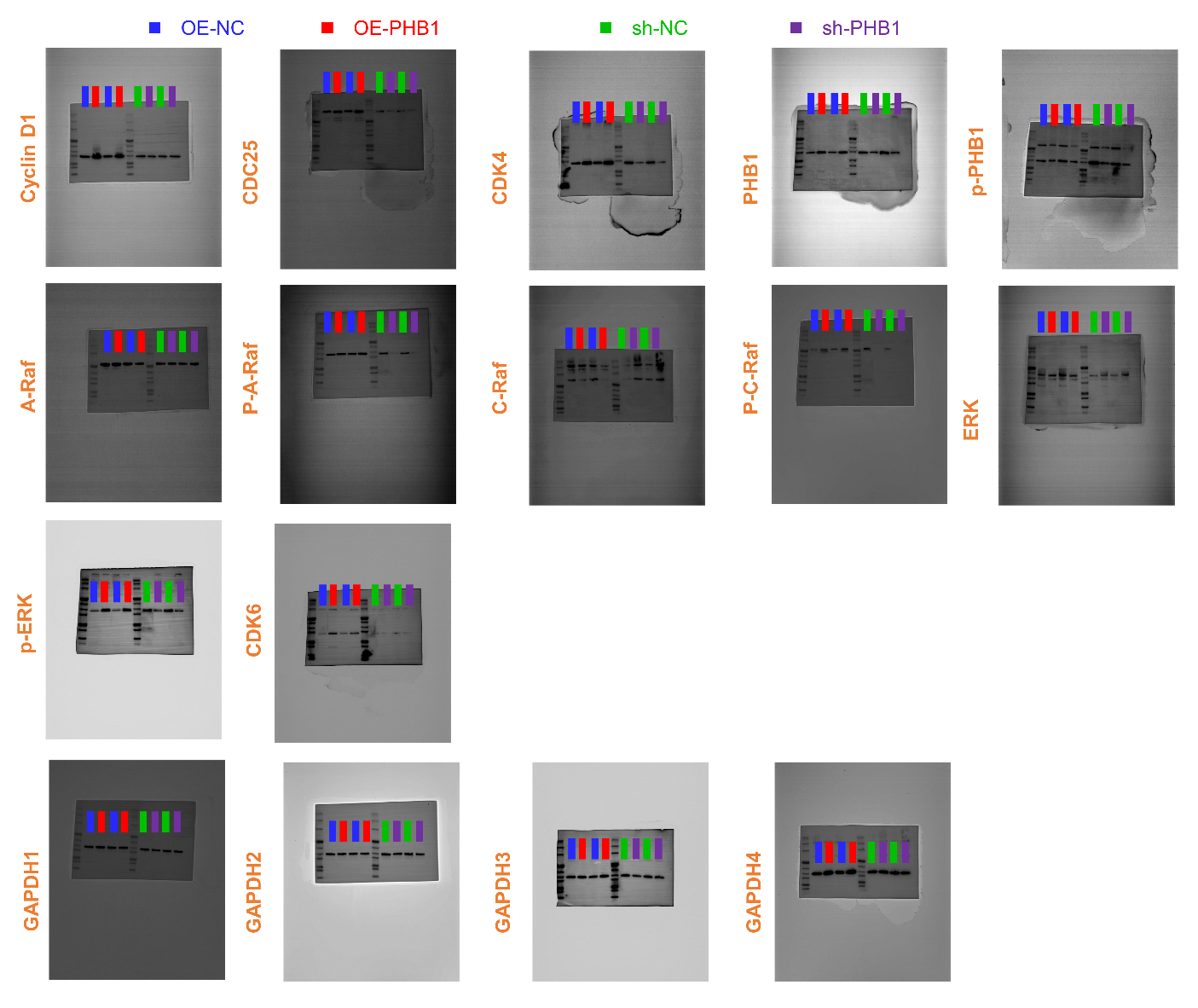


**FIGURE S12** Western blot analysis of PHB1, p‑PHB1, p‑A‑Raf, A‑Raf, p‑C‑Raf, C‑Raf, p‑ERK1/2, ERK1/2, CDK4, Cyclin D1 and GAPDH in OE‑NC, OE‑PHB1, sh‑NC, and sh‑PHB1 groups (sample 1 and 2). Each lane corresponds to the indicated group in order.

**FIGURE S13**

**
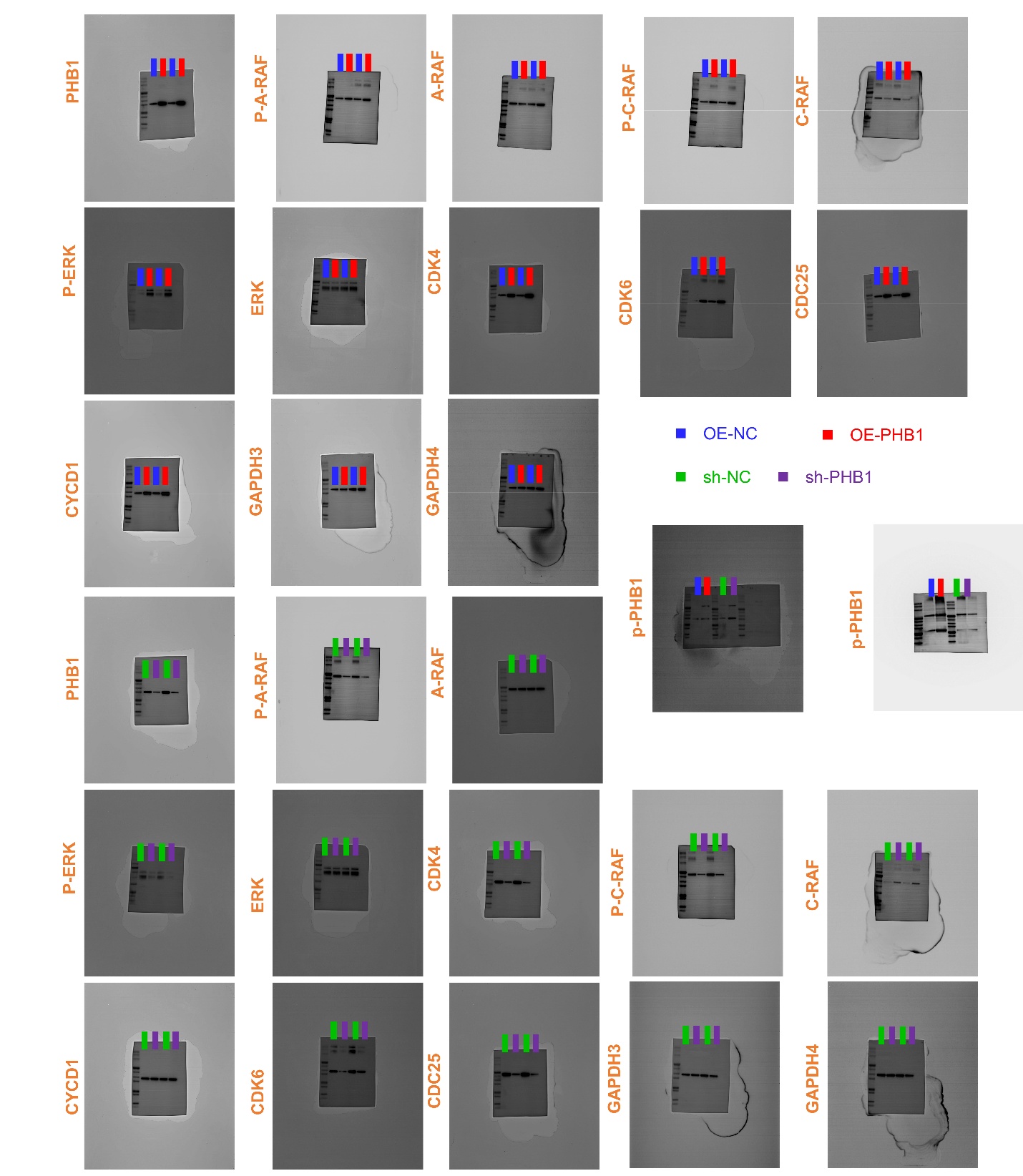
**

**FIGURE S13** Western blot analysis of PHB1, p‑PHB1, p‑A‑Raf, A‑Raf, p‑C‑Raf, C‑Raf, p‑ERK1/2, ERK1/2, CDK4, CDK6, Cyclin D1, CDC25 and GAPDH in OE‑NC, OE‑PHB1, sh‑NC, and sh‑PHB1 groups (sample 3 and 4). Each lane corresponds to the indicated group in order.

**FIGURE S14**


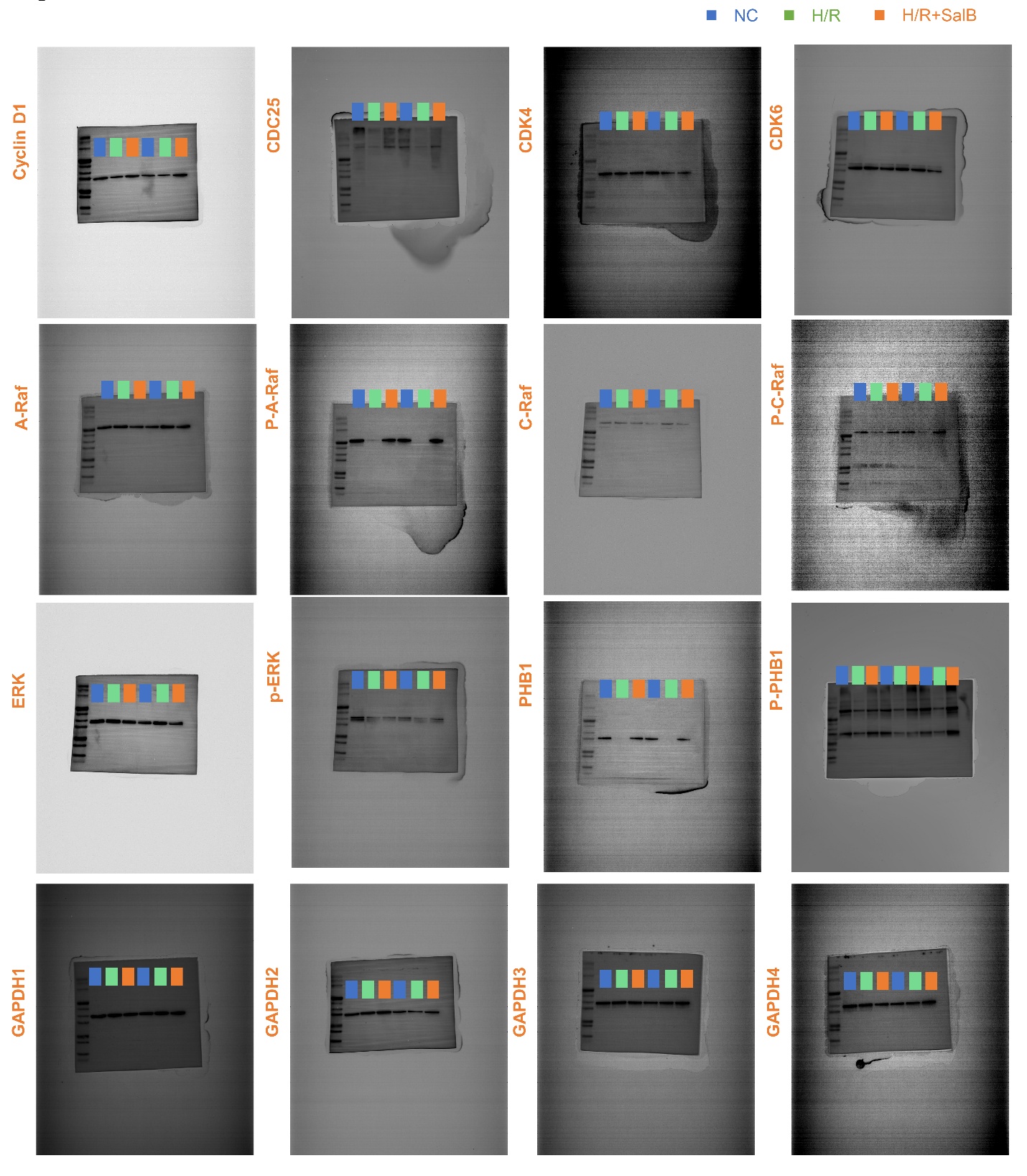


**FIGURE S14** Western blot analysis of PHB1, p‑PHB1, p‑A‑Raf, A‑Raf, p‑C‑Raf, C‑Raf, p‑ERK1/2, ERK1/2, CDK4, CDK6, Cyclin D1, CDC25 and GAPDH in NC, H/R, and H/R+SalB groups. Each lane corresponds to the indicated group in order.

**FIGURE S15**


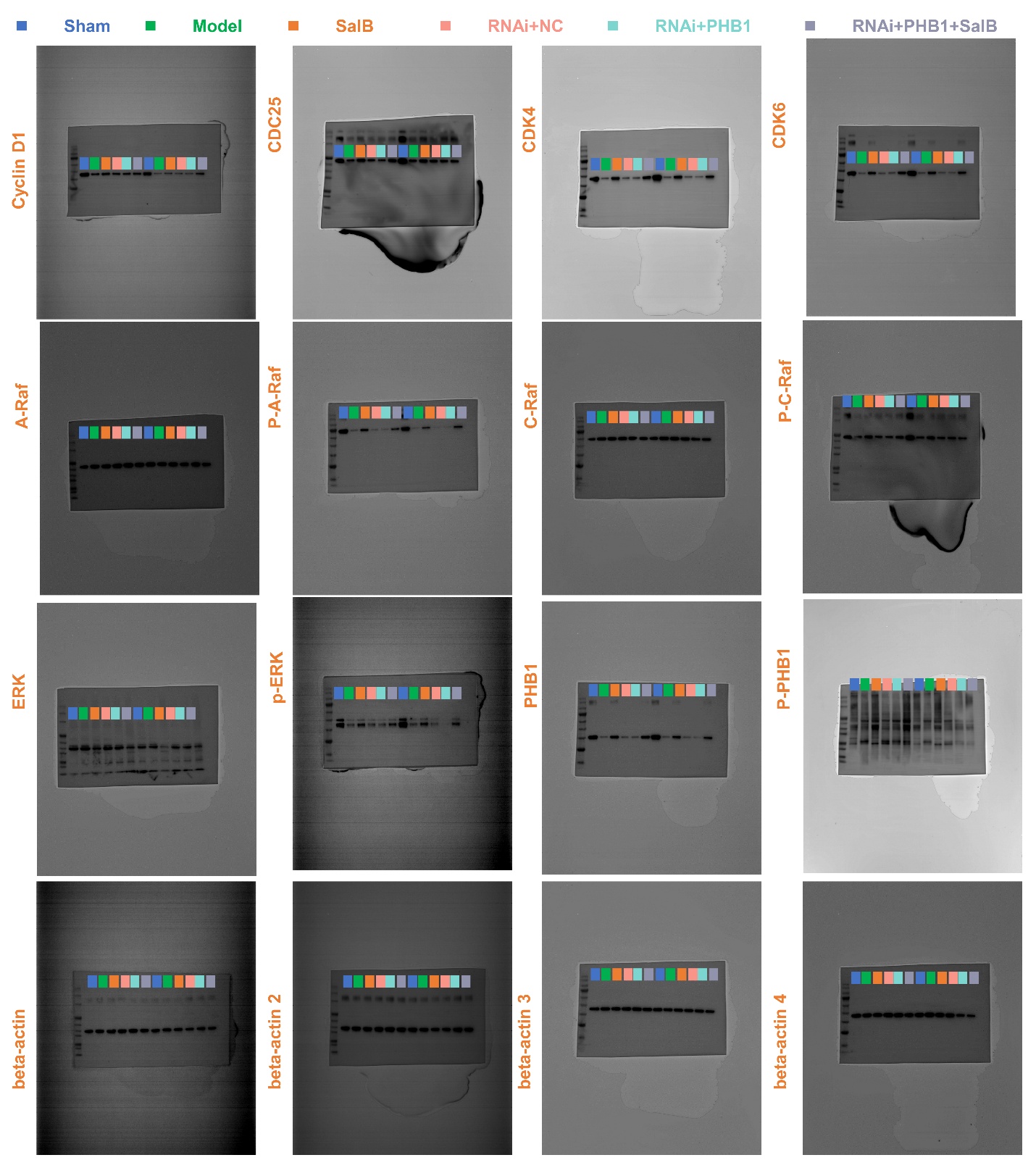


**FIGURE S15** Western blot analysis of PHB1, p‑PHB1, p‑A‑Raf, A‑Raf, p‑C‑Raf, C‑Raf, p‑ERK1/2, ERK1/2, CDK4, CDK6, Cyclin D1, CDC25 and GAPDH in Sham, Model, SalB, RNAi+NC, RNAi+PHB1, and RNAi+PHB1+SalB groups. Each lane corresponds to the indicated group in order.

**FIGURE S16**


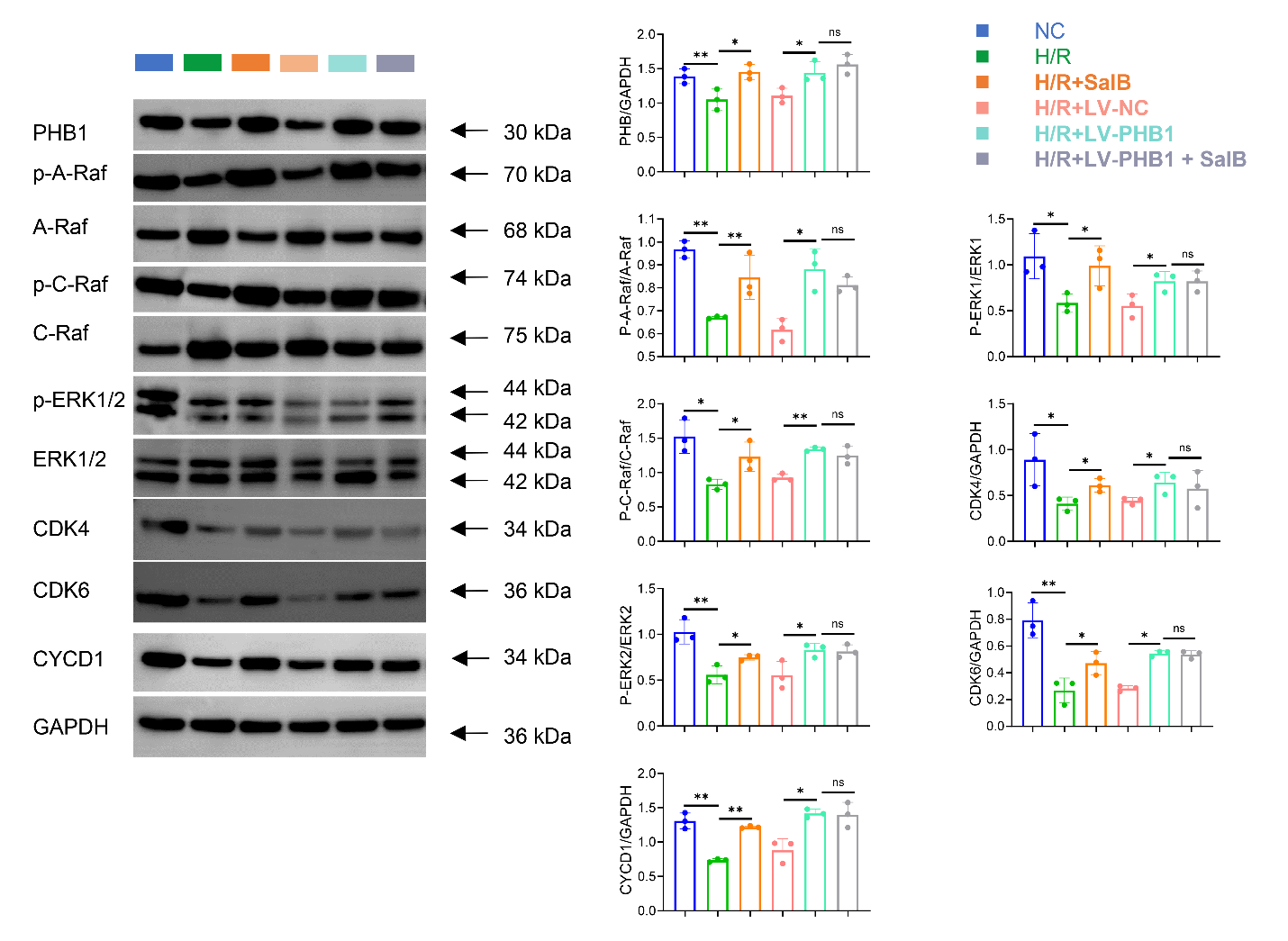


**FIGURE S16** Expression levels of PHB1, p‑PHB1, p‑A‑Raf, A‑Raf, p‑C‑Raf, C‑Raf, p‑ERK1/2, ERK1/2, CDK4, CDK6, and Cyclin D1 in human cardiomyocytes with myocardial infarction‑related heart failure (n=3). Each lane corresponds to NC, H/R, H/R+SalB, H/R+LV‑NC, H/R+LV‑PHB1 and H/R+LV‑PHB1+SalB groups in order.

**FIGURE S17**

**
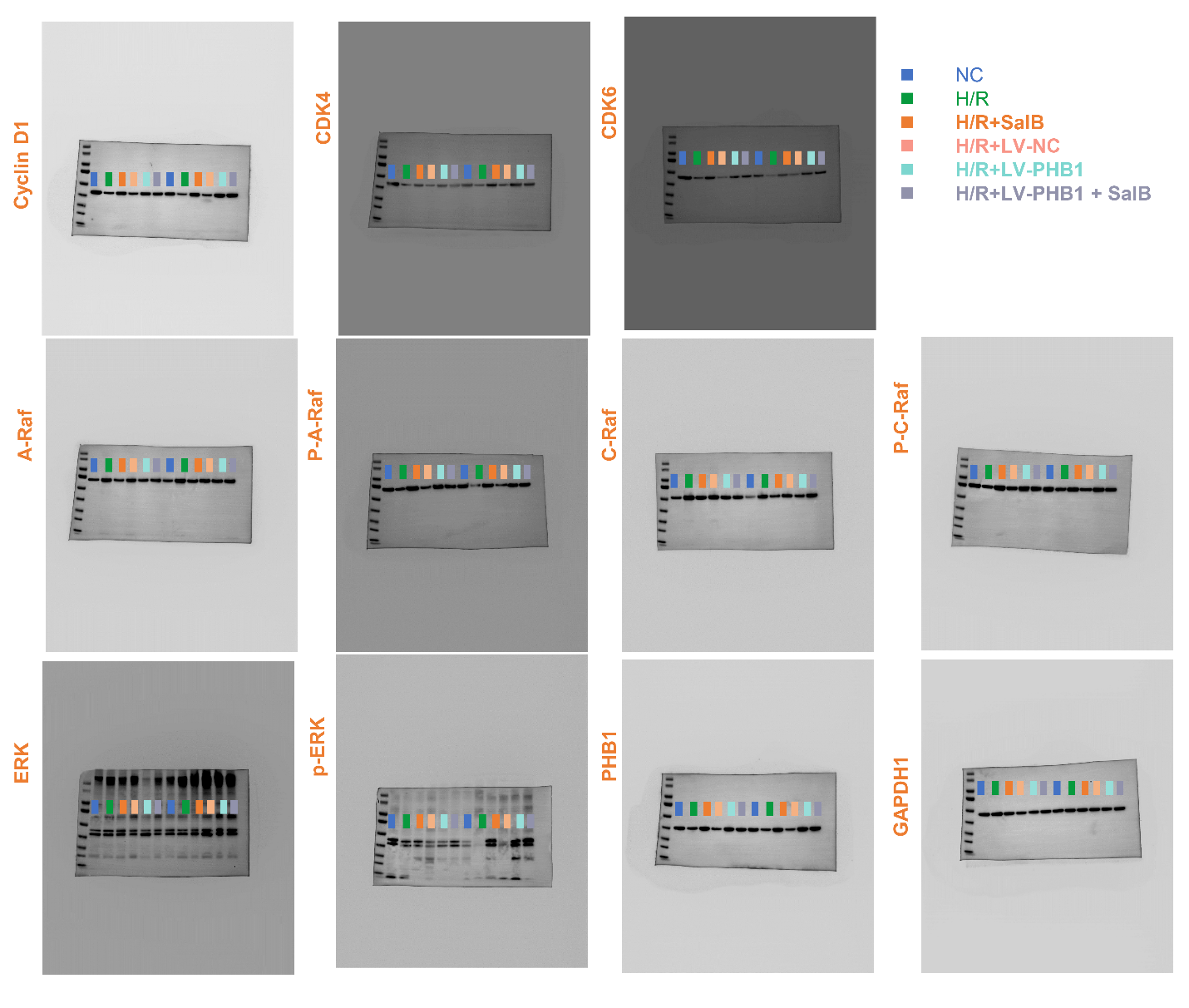
**

**FIGURE S17** Western blot analysis of PHB1, p‑PHB1, p‑A‑Raf, A‑Raf, p‑C‑Raf, p‑ERK1/2, ERK1/2, CDK4, CDK6, Cyclin D1, and GAPDH in human cardiomyocytes. Each lane corresponds to NC, H/R, H/R+SalB, H/R+LV‑NC, H/R+LV‑PHB1, and H/R+LV‑PHB1+SalB groups in order.
